# Productive and metabolomic consequences of arginine supplementation in sows during different gestation periods in two different seasons

**DOI:** 10.1186/s40104-024-01079-4

**Published:** 2024-09-19

**Authors:** Sara Virdis, Diana Luise, Federico Correa, Luca Laghi, Norma Arrigoni, Roxana Elena Amarie, Andrea Serra, Giacomo Biagi, Clara Negrini, Francesco Palumbo, Paolo Trevisi

**Affiliations:** 1 Department of Agricultural and Food Sciences, University of Bologna, Viale G. Fanin 44, 40127 Bologna, Italy; 2 Istituto Zooprofilattico Sperimentale Della Lombardia E Dell’Emilia Romagna (IZSLER) “Bruno Ubertini”, Via Bianchi, 9, 25124 Brescia, Italy; 3 Department of Agriculture, Food and Environment, University of Pisa, Via del Borghetto 80, 56124 Pisa, Italy; 4 Department of Veterinary Medicine, University of Bologna, Via Tolara Di Sopra 50, 40064 Ozzano Dell’Emilia (BO), Italy

**Keywords:** Colostrum, Feces, Microbiota, Piglets’ birth weight, Stillborn, Urine

## Abstract

**Background:**

The prolificacy of sows (litter size at birth) has markedly increased, leading to higher post-natal mortality. Heat stress can exacerbate this issue. Arginine plays an important role in several physiological pathways; its effect on gestating sows can depend on the period of supplementation. This study evaluated the effects of arginine supplementation on the productive performance and physiological status of sows during different gestation periods and seasons, using a multi-omics approach.

**Methods:**

A total of 320 sows were divided into 4 groups over 2 seasons (warm/cold); a control group (CO) received a standard diet (including 16.5 g/d of arginine) and 3 other groups received the standard diet supplemented with 21.8 g/d of arginine (38.3 g/d of arginine) either during the first 35 d (Early35), the last 45 d (Late45) or throughout the entire gestation period (COM). The colostrum was analyzed for nutritional composition, immunoglobulins and metabolomic profile. Urine and feces were analyzed on d 35 and 106 for the metabolomic and microbial profiles. Piglet body weight and mortality were recorded at birth, d 6, d 26, and on d 14 post-weaning.

**Results:**

Interactions between arginine and season were never significant. The Early35 group had a lower percentage of stillborn (*P* < 0.001), mummified (*P* = 0.002) and low birthweight (LBW) piglets (*P* = 0.02) than the CO group. The Late45 group had a lower percentage of stillborn piglets (*P* = 0.029) and a higher percentage of high birthweight piglets (HBW; *P* < 0.001) than the CO group. The COM group had a higher percentage of LBW (*P* = 0.004) and crushed piglets (*P* < 0.001) than the CO group. Arginine supplementation modifies the metabolome characterization of colostrum, urine, and feces. Creatine and nitric oxide pathways, as well as metabolites related to microbial activity, were influenced in all matrices. A slight trend in the beta diversity index was observed in the microbiome profile on d 35 (*P* = 0.064).

**Conclusions:**

Arginine supplementation during early gestation reduced the percentage of stillborn and LBW piglets, while in the last third of pregnancy, it favored the percentage of HBW pigs and reduced the percentage of stillbirths, showing that arginine plays a significant role in the physiology of pregnant sows.

**Supplementary Information:**

The online version contains supplementary material available at 10.1186/s40104-024-01079-4.

## Introduction

During the last decade, prolificacy in terms of litter size at birth has markedly increased in sows [1]. However, their uterine capacity is not commensurate with the current litter size, in terms of both physical space and the availability of nutrients [2]. This leads to a greater number of low birthweight (LBW) piglets [3], a trait positively correlated with litter size [4]. These factors cause higher pre- and post-natal mortality, impacting the revenue of farmers, especially during the warm season. In fact, heat stress (HS) leads to a series of adaptive metabolic and behavioral changes, including a reduction in the daily feed intake (FI), alterations in follicular development and oocyte quality, decreased luteal size and compromised embryo development [5]. In addition, oxidative stress, exacerbated by HS during gestation, leads to a decrease in colostrum, and milk quality and yield [6].

In this scenario, some nutrients can play a pivotal role. Amino acids (AAs) serve both as building blocks for proteins, and as essential functional and signaling molecules in the body. They play a crucial role in supporting animal health and performance [7, 8]. Clarifying the nutrient requirements of the sows can minimize the aforementioned negative aspects and can optimize the sow’s genetic potential. In particular, arginine (Arg) is involved in several biological processes, including immunity and skeletal muscle function, energy and protein metabolism, insulin sensitivity, and growth hormone production [9]. Moreover, its involvement in protein synthesis and in the activation of the mTOR (mechanistic Target of Rapamycin) [10] could have a positive effect by reducing the loss in loin depth which occurs when sows are affected by HS. It plays a crucial role in maintaining the intestinal microbial balance by serving as a source of microbial carbon, nitrogen, and energy [11, 12]. This, in turn, affects the growth, differentiation, and metabolic activity of the microbes. Thus, the supplementation of AAs from the Arg family, such as proline and glutamine, can play a pivotal role in the development of the placenta and fetuses [13], and has been considered a conditionally essential AA for sows by the National Research Council (NRC) since 2012 [14]. According to the NRC [14], Arg requirements for gestating sows depend on period of gestation, class of parity, body weight (BW) at insemination, and estimated litter size. Based on these variables, the requirements of Arg are 6.35 g/d, 5.5 g/d, and 4.68 g/d for the second, third, and fourth classes of parity, respectively. However, when considering the increase in litter size due to the evolution of the breed line which has occurred in recent years [1], Arginine requirements may be greater than those estimated by the NRC [14], as suggested by a recent meta-analysis by Virdis et al. [15]. Recently, limited data have become accessible for estimating Arg requirements in gestating sows in order to maximize their performance; however, the role of Arg in addressing the issues related to HS remains unclear.

Arginine has had different effects on gestating sows based on the timing of supplementation during gestation. Arginine supplementation in early gestation can regulate angiogenesis and vascular development, improving placental efficiency, and enhancing the supply and the availability of nutrients and oxygen from the sow to the fetus [16, 17]; it also seems to increase the number of fetuses [18]. In late gestation, Arg supplementation increases the birth weight of piglets and improves litter uniformity [19, 20]. However, the impact of Arg on sow prolificacy is still under debate, with some studies suggesting that it could negatively affect litter uniformity [21]. Therefore, taking this background into account, the aim of this study was to evaluate the effects of Arg supplementation on the productive and physiological status of sows during different stages of gestation and different seasons. The present study adopted a multi-omics approach, coupled with fecal microbial analysis, to investigate the effect of Arg on sow physiology, using metabolomics to analyze colostrum, urine and feces.

## Material and methods

### Experimental design

The study enrolled a total of 320 sows (Topigs TN70) which had been fed the same diet and reared under the same conditions before the start of the study. At the beginning of gestation, the sows were divided into 4 experimental groups (80 sows/group), based on BW and parity: 1) Control (CO) group, fed with a standard gestation diet; 2) Early35 group, fed with the CO diet supplemented with 21.8 g/d of top Arg for the first 35 d of gestation; 3) Late45 group, fed with the CO diet supplemented with 21.8 g/d of on top Arg for the last 45 d of gestation; 4) COM group, fed with the CO diet supplemented with 21.8 g/d of on top Arg throughout the entire gestation period. Furthermore, the study was carried out during 2 distinct periods, representing different seasons; the warm season covered the pregnancy phase from July to October, and the cold season covered it from November to January. The distribution of the sows during the 2 seasons for the CO, Early35, Late45 and COM groups corresponded to 40, 37, 40 and 31 sows, respectively during the warm season and 40, 42, 39 and 48 sows, respectively during the cold season.

The sows were classified into three different classes based on their parity order: old (7^th^ and 8^th^ order), mid (4^th^, 5^th^ and 6^th^ order) and young (2^nd^ and 3^rd^ order). Primiparous sows were not included in this study.

During gestation, the sows were reared in groups of approximately 70 animals and were fed using an automatic feeder Schauer Compident station (Schauer, Austria) endowed with an animal identification system; all the sows were fed according to a specific feeding curve depending on the season. During the warm season, the sows received a daily dose of 2.5 kg between d 1 and 41, 2.0 kg between d 42 and 109 and 2.7 kg from d 110 until farrowing; during the cold season, they received 2.4 kg between d 1 and 84 and 2.8 kg from d 85 until farrowing. The basal diet formulation and calculated composition are reported in Table 1. The Arg content of the CO diet was equal to 16.5 g/d.

**Table 1:** Basal diet formulation fed to sows during gestation and lactation

Ingredients	Gestation	Lactation
Barley, %	15.00	15.00
Corn (grounded), %	14.98	11.50
Corn 8% CP, %	10.80	15.00
Triticale, %	10.00	10.00
Wheat, %	10.00	–
Wheat bran, %	7.33	3.17
Sunflower meal 35% CP, %	7.00	3.00
Beet pulp, %	6.00	3.00
Sorghum, %	5.00	–
Sunflower oil, %	3.00	1.67
Grease, %	2.00	1.67
Oat, %	1.67	3.17
Soybean 48% CP, %	1.50	15.10
Sugar cane molasses, %	1.50	2.33
Soybean, %	1.33	7.50
Soybean hulls, %	–	1.67
Bakery meal, %	–	2.50
Calcium carbonate, %	0.87	0.97
Monocalcium phosphate, %	0.48	0.65
Sodium chloride, %	0.47	0.38
Mineral mix, %	0.40	0.40
Magnesium oxide, %	–	0.20
Liquid lysine 50%, %	0.39	0.65
Liquid choline 75%, %	0.10	0.13
Threonine, %	0.08	0.16
HiPhosphate, %	0.10	0.10
Methionine, %	–	0.04
Tryptophan, %	–	0.02
Vitamine E, %	–	0.02
Chemical composition		
Dry matter, %	87.79	87.80
Protein, %	12.27	17.97
Lipids, %	7.5	6.80
Crude fiber, %	5.26	5.01
Ash, %	4.74	5.65
Calcium, %	0.7006	0.7948
Phosphorus, %	0.4512	0.4978
P.AS.SUS, %	0.3112	0.3550
NDF, %	2.422	14.1506
Linoleic acid, %	3.0429	2.9498
Starch, %	41.0527	33.3894
Sugar, %	3.5196	5.0205
NA, %	0.2211	0.1995
K, %	0.5734	0.8838
Cl, %	0.3887	0.3601
Lys, %	0.6243	1.1727
Met, %	0.2488	0.3458
Met + Cys, %	0.4799	0.6443
Trp, %	0.137	0.2309
Thr, %	0.4769	0.7795
Arg, %	0.6876	1.1165
SID Lys, ED/Mcal	2.20	4.42
ED pigs, kcal/kg	3.300	3.363
EM pigs, kcal/kg	3.188	3.210
EN pigs, kcal/kg	2.461	2.365

The inclusion of an additional 21.8 g/d of Arg, on top of that in the CO diet was chosen to reach a total amount of 38.3 g/d of Arg, based on the results obtained by the meta-analysis carried out by Virdis et al. [15]. The sows had ad-libitum access to water during both gestation and lactation.

One week before farrowing, the sows were moved into the farrowing unit in which each feeder was served by an independent duct. The feed distribution in the farrowing unit was carried out using a Spotmix (Schauer, Austria) equipped with a small tank which allowed providing the Arg to the sows belonging to the Late45 and COM groups, even during the last week of gestation.

### Collection of samples

The sows were weighed on d 5 ± 3 and on d 106 ± 4 of gestation when they were moved to the farrowing unit. On d 35 of gestation (the last day of Arg supplementation for the Early35 group), a sample of urine was collected from 14 sows [7 sows supplemented with Arg (Early35 and COM) and 7 sows not supplemented with Arg on d 35 (CO and Late45)] over a 1.5–2.5-h timespan after their last meal. Urine was collected in a sterile tube and immediately frozen in liquid nitrogen for metabolomic analysis; it was collected only during the warm season.

On d 35 and d 106, a total of 64 fecal samples/timepoint (16 sows/group, 8 sows/season/group) were collected from the sows. Feces were collected in a sterile tube and immediately frozen in liquid nitrogen for short-chain fatty acid (SCFA; only d 106), metabolomic (only d 106) and microbial analyses.

At farrowing, after the birth of the first piglet and within 4 piglets farrowed, a total of 96 samples of colostrum (24 samples/group, 12 samples/season/group) were obtained by milking all functional teats as reported by Luise et al. [22] to characterize the colostrum composition and analyze the concentration of immunoglobulins (Igs) A, G and M. Moreover, from each sample, an aliquot of colostrum was snap-frozen in liquid nitrogen and stored at –80 °C until the metabolomic analysis.

At farrowing, the numbers of total piglets born, born alive and stillborn, mummified, and crushed were recorded for each sow. The number of stillborn and mummified piglets was expressed as a percentage of the total number of piglets born, while the number of crushed piglets was expressed as a percentage of the number of piglets born alive. At farrowing (d 0), the piglets were identified with a numbered ear tag and weighed (after colostrum consumption), and the coefficient of variation (CV) of the birth weight within the litter was calculated. From 52 litters per group, the BW at birth of all piglets born (including stillborn, mummified, and crushed) was collected. The pigs born alive were classified according to their birth BW into three classes: low body weight (LBW < 1 kg), normal BW (1.0 kg ≤ NBW ≤ 1.7 kg) or high BW (HBW > 1.7 kg). Cross-fostering of piglets was carried out within 48 h after farrowing among sows belonging to the same experimental group in order to standardize litter size, to match the sow’s rearing capacity with litter size, and to ensure that all the piglets could access a functional teat. All the male piglets were castrated within the first week of life, as in the usual routine breeding practices and caudectomy was not performed. The piglets were then individually weighed at 6 d of life (d 6 ± 1), at weaning (d 26 ± 2), and at 14 d post-weaning (d 14 pw) and the relative average daily gain (ADG) was calculated.

Mortality rate and the number of removals were recorded at each timepoint based on the experimental groups.

### Colostrum chemical composition and immunoglobulins concentration

Colostrum samples were diluted 1:1 with water and were then analyzed using the Milkoscan FT2 to determine the composition in terms of fat, protein, lactose, casein and urea [23]. The colostrum samples were prepared for metabolomic and Ig concentration analysis as reported by Picone et al. [24]. Briefly, 1.5 mL of colostrum was diluted 1:1 with pure water and 0.02% of sodium azide. The diluted samples were centrifuged at 4 °C for 30 min at 1,500 × *g* to obtain the defatted colostrum. The Ig concentration analysis was carried out using ELISA (enzyme-linked immunosorbent assay), using the goat anti-pig IgA/IgM/IgG-affinity purified and goat anti-pig IgA/IgM/IgG HRP conjugate antibodies (Bethyl Laboratories, Montgomery, TX, USA) and Pig Immunoglobulin Reference Serum RS107-4 (Bethyl Laboratories, Montgomery, TX, USA) as specific standard. Absorbance was measured at 405 nm using a plate reader (Multiskan™ FC Microplate Photometer–Thermo Fisher Scientific). The concentration values, expressed as µg/mL, were calculated using a 5-point parametric curve. The colostrum defatted samples were previously diluted 1:50,000, 1:10,000, and 1:500,000 for IgA, IgM, and IgG analysis as reported by Luise et al. [25].

### NMR spectroscopy analysis for identifying metabolomes from urine, feces, and colostrum

The metabolomic profiles of the urine, feces, and defatted colostrum samples were investigated following the procedure described by Brugaletta et al. [26].

### Analysis of the short-chain fatty acids and ammonia present in the feces

A total of 64 samples of feces from d 106 were analyzed for ammonia and SCFAs (lactate, acetate, propionate, isobutyrate, butyrate, isovalerate, and valerate).

For the determination of ammonia in fecal samples, 1 g of feces was thawed, and was then diluted with deionized water at a weight/volume ratio of 1:10. After vertexing, the samples were centrifuged at 7,000 r/min at 4 °C for 10 min. The ammonia content of the fecal supernatant was determined using an enzymatic colorimetric assay according to the manufacturer’s protocol (Urea/BUN-Color; BioSystems S.A., Barcelona, Spain); the results were expressed as μmol/g feces.

The analyses of the SCFAs in the sow feces were carried out by adapting the method of Trevisi et al. [27], using high-performance liquid chromatography (HPLC).

### Microbiome analysis

Microbial DNA was extracted from the feces samples using the FastDNA™ Spin Kit for Soil (MP Biomedicals Europe, LLC). The DNA quality and quantity were determined by quantification using Nanodrop, and an agarose gel load and run. The DNA was then used to characterize the sows’ microbial profile by sequencing the V3–V4 hypervariable regions of gene 16S rRNA as described by Luise et al. [28]. Briefly, the standard protocol for the MiSeq Reagent Kit v3 was used to prepare the libraries. The libraries were sequenced on the MiSeq platform (Illumina Inc., San Diego, CA, USA). Bioinformatic analysis was carried out using the DADA2 pipeline [29], and taxonomy was assigned using the Silva database (release 138.1) [30].

### Statistical and bioinformatic analysis

Statistical analyses were carried out using RStudio v4.3 (RStudio, PBC, Boston, MA, USA) with “*jmv”* [31], “*car”* [32], “*emmeans”* [33], and “*lme4”* [34]. The data were fitted using a linear mixed model in which the experimental diet (CO vs. Early35 vs. Late45 vs. COM), the season (warm vs. cold), and the class of parity (old vs. mid vs. young) were included as factors; while, regarding piglets’ growing performance, the age of the piglets when being weighed, litter size and BW at the previous timepoint were included as covariates. The sow was included as a random factor for the analysis of the piglets’ growing performance (e.g., piglet weight at d 0, 6, and 26, and the relative ADG). The interaction between experimental diets and seasons was tested, and was then removed when not significant. The distribution of data was tested using the Shapiro-test and the “*LaplacesDemon*” [35] package, and was corrected when it was not normal. Comparisons between dietary groups and seasons were tested using a post-hoc test (Tukey test).

The results were expressed as least-square means and standard error of the mean (SEM). A difference was declared significant when *P* ≤ 0.05 and marginally significant when 0.05 < *P* ≤ 0.10.

For the mortality and the removal rate, a Fisher test was carried out with contrasts calculated using the “*RVAideMemoire*” [36] package on RStudio v4.3 (RStudio, PBC, Boston, MA, USA).

The statistical analysis of the metabolomic data involved 2 parts. First, metabolomic datasets were analyzed with a multivariate approach using MetaboAnalyst 6.0 [37]; the metabolites were then analyzed using an ANOVA model on RStudio v4.3 (RStudio, PBC, Boston, MA, USA). In the multivariate analysis, the data were normalized using sum and log transformation to account for systematic differences between samples. After the normalization data were analyzed using partial least squares-discriminant analysis (PLS-DA) on MetaboAnalyst 6.0, the variable importance projection (VIP) scores were determined for diet and season. For the univariate analyses, the data of the colostrum and feces were fitted using a linear mixed model which included diet and season as fixed factors. Since urine was collected on d 35, only 2 dietary groups were present at that timepoint: the CO group (samples from CO and Late45 groups) and the ARG group (samples from Early35 and COM groups).

Regarding the microbial data, the statistical analyses of alpha diversity indices (Chao, Shannon, InvSimpson indices), beta diversity (calculated as Bray Curtis distance matrix), and taxonomic composition were carried out with RStudio v4.3 using “*phyloseq*” [38], “*vegan*” [39], “*lme4*” [34] packages. Alpha diversity indices were analyzed for both timepoints (d 35 and d 106 of gestation) using an ANOVA model (“Adonis” procedure) which included experimental group, season, and class of parity as fixed factors. For beta diversity, the Bray Curtis distance matrix was calculated and plotted using a non-metric multidimensional scaling plot; the differences were tested using a PERMANOVA (Adonis) model with 9,999 permutations, including diet, season, and their interaction as factors. For the differential analysis of the taxa, the LEfSe algorithm [40] was used at the genus level. A linear discriminant analysis (LDA) score > 3 and *P*_adj _< 0.05 were considered to identify taxa differentially expressed among the dietary groups and seasons.

## Results

Since the effect of season was not the main objective of the present study, only the most significant interactions between diet and season will be discussed in the following sections. However, the results related to the season will be provided in order to guarantee complete information to the reader.

### Growth performance of sows during gestation

A total of three sows, one from each of the Early35, Late45 and COM groups, were removed from the study as a result of aborting.

Gestation length did not differ between the experimental groups, having an average of 116 d.

Table 2 reports the effects of Arg and season on sow growth performance. The interaction between group and season was never significant. Bodyweight on d 0 did not differ among the experimental groups, while on d 106, diet affected the BW (*P* = 0.023). In addition, it tended to affect the ADG from d 0 to d 106 (*P* = 0.056). In detail, the COM group had a higher BW and ADG than the CO group (298 vs. 292 kg, *P* = 0.003; 503 vs. 445 g/d, *P* = 0.040, respectively). No differences were observed in the other groups.

**Table 2:** Effect of Arg supplementation and seasons on growing performance of sows during gestation

Items	Diet^1^					Season^2^			*P*-value	
	**CO**	**Early35**	**Late45**	**COM**	**SEM**	**Warm**	**Cold**	**SEM**	**Diet**	**Season**
BW d 0, kg	245	249	249	249	2.72	241^Z^	254^Y^	1.94	0.704	< 0.001
BW d 106, kg	292^B^	295^AB^	296^AB^	298^A^	1.46	299^Y^	292^Z^	1.06	0.023	< 0.001
ADG, g/d	445^B^	470^AB^	486^AB^	503^A^	15.28	528^Y^	423^Z^	10.9	0.056	< 0.001

The interaction between the diet and the season was tested and removed from the statistical model if not statistically significant

Means within a row with different superscripts differ for diets’ contrasts ^AB^ *P* < 0.05 and ^ab^ *P* < 0.10; for seasons’ contrast ^YZ^ *P* < 0.05 and ^yz^ *P* < 0.10

*BW*, Body weight; *ADG *(BW d 106 − BW d 0)/106

^1^ A total of 320 sows were divided into 4 experimental groups: CO, fed with a basal diet; Early35, fed with the CO diet with 21.8 g/d on-top Arg during the first 35 d of gestation; Late45, fed with the CO diet with 21.8 g/d on-top Arg during the last 45 d of gestation; COM, fed with the CO diet with 21.8 g/d on-top Arg during all gestation

^2^ The trial was performed during 2 seasons: warm, sows performed gestation between July and September; cold, sows performed gestation between November and January

### Litter and piglet performance

Table 3 reports the effects of Arg and season on sow performance at farrowing. The interaction between group and season was never significant. The total number of piglets born and the number of piglets born alive were not affected by diet. The percentage of mummified piglets was higher in the COM group as compared with the Early35 (*P* = 0.002) and the Late45 (*P* = 0.049) groups. The percentage of stillborn piglets did not differ between the CO and the COM groups. The CO group had a higher stillborn percentage than the Early35 group (10.39% vs. 8.11%, *P* < 0.001) and the Late45 (10.39% vs. 9.04%, *P* = 0.029) groups, whereas this percentage was lower in the Early35 as compared with the COM group (8.11% vs. 9.70%, *P* = 0.004). The COM group showed the highest percentage of crushed piglets at birth (*P* < 0.001). Furthermore, some performance parameters of the sows at farrowing were influenced by season. In brief, the total number of piglets born (*P* = 0.003) and the percentage of mummified piglets (*P* < 0.001) was lower in the warm season. Moreover, it tended to reduce the percentage of stillborn piglets (*P* = 0.081) while it increased the percentage of crushed piglets (*P* < 0.001).

**Table 3:** Effect of Arg supplementation and seasons on the performance of sows at farrowing

Items	Diet^1^					Season^2^			*P*-value	
	**CO**	**Early35**	**Late45**	**COM**	**SEM**	**Warm**	**Cold**	**SEM**	**Diet**	**Season**
Total born, n	18.0	17.4	17.7	18.7	0.39	17.3^Z^	18.5^Y^	0.28	0.108	0.003
Total born alive, n	15.5	15.6	15.6	16.3	0.35	15.5	16.0	0.25	0.400	0.106
Mummified, %	1.86^AB^	1.43^B^	1.62^B^	2.17^A^	0.15	1.27^Z^	2.40^Y^	0.11	0.002	< 0.001
Stillborn, %	10.39^A^	8.11^B^	9.04^B^	9.70^A^	0.33	8.98^y^	9.57^z^	0.12	< 0.001	0.081
Crushed, %	1.74^B^	1.74^B^	1.82^B^	3.15^A^	0.18	2.39^Y^	1.74^Z^	0.11	< 0.001	< 0.001

Means of stillborn and mummified were expressed as a percentage of the total born piglets. Means of crushed were expressed as a percentage of the total born alive

The interaction between the diet and the season was tested and removed from the statistical model if not statistically significant

Means within a row with different superscripts differ for diets’ contrasts ^AB^ *P* < 0.05 and ^ab^ *P* < 0.10; for seasons’ contrast ^YZ^ *P* < 0.05 and ^yz^ *P* < 0.10

^1^ A total of 320 sows were divided into 4 experimental groups: CO, fed with a basal diet; Early35, fed with the CO diet with 21.8 g/d on-top Arg during the first 35 d of gestation; Late45, fed with the CO diet with 21.8 g/d on-top Arg during the last 45 d of gestation; COM, fed with the CO diet with 21.8 g/d on-top Arg during all gestation

^2^ The trial was performed during 2 seasons: warm, sows performed gestation between July and September; cold, sows performed gestation between November and January

Table 4 reports the effects of Arg and season on the growing performance of piglets from birth until 14 d post-weaning. There was no significant interaction between group and season regarding the measurements collected on d 0.

**Table 4:** Effect of Arg supplementation and season on the growing performance of piglets from birth to 14 d after weaning

**Items**	**Diet**^1^					**Season**^2^			***P***-value	
	**CO**	**Early35**	**Late45**	**COM**	**SEM**	**Warm**	**Cold**	**SEM**	**Diet**	**Season**
d 0										
BW of total born^3^, g	1,366	1,375	1,366	1,319	26.48	1,329^z^	1,384^y^	19.85	0.420	0.054
CV of BW of total born^4^, g	22.9	22.4	22.1	23.4	0.96	21.8^z^	23.6^y^	0.721	0.740	0.087
Total litter weight^5^, g	24,177	24,073	24,278	23,287	484	23,508^z^	24,399^y^	362	0.436	0.092
BW of born alive^6^, g	1,411	1,411	1,406	1,351	26.4	1,355^Z^	1,434^Y^	19.75	0.289	0.006
CV of BW of born alive^7^, g	19.1	19.4	19.0	20.0	0.79	19.5	19.2	0.59	0.798	0.745
Alive litter weight^8^, g	21,623	22,180	21,856	21,038	562	21,105^z^	22,244^y^	421	0.518	0.063
LBW^9^, %	10.07^B^	8.41^C^	11.70^A^	12.94^A^	1.04	12.55^Y^	9.12^Z^	1.17	< 0.001	< 0.001
NBW^10^, %	68.8	64.8	63.5	64.2	2.83	67.1	63.5	2.12	0.529	0.244
HBW^11^, %	15.96^BC^	16.95^B^	18.73^A^	15.03^C^	1.03	14.30^Z^	19.30^Y^	1.03	< 0.001	< 0.001
d 6										
BW^12^, g	2,438	2,446	2,485	2,450	32.2	2,456	2,453	23.15	0.747	0.937
ADG d 0– 6^13^	147	150	155	144	5.28	145	153	3.82	0.502	0.131
d 26										
BW^14^, g	6,467	6,429	6,463	6,319	163	6,146^Z^	6,692^Y^	73.2	0.204	0.004
ADG d 6– 26^15^	226	227	222	218	6.39	216	230	6.58	0.567	0.102
ADG d 0– 26	203	206	204	197	5.91	192^Z^	212^Y^	3.04	0.403	0.006
d 14 post-weaning										
BW^16^, g	9,430	9,749	9,531	9,562	152	9,122^Z^	10,015^Y^	134	0.385	< 0.001
ADG d 26–d 14 pw	172	187	179	177	8.54	151^Z^	207^Y^	7.04	0.543	< 0.001
ADG d 0–d 14 pw	185	197	194	187	5.40	176^Z^	205^Y^	4.81	0.098	< 0.001

Parity order was included as covariate for the all the BW, CV, litter weights and for the body categories percentages: old (7^th^ and 8^th^ order); mid (4^th^, 5^th^ and 6^th^ order); young (2^nd^ and 3^rd^ order). Parity order affected the CV of total born^4^ (old vs. young, *P* = 0.027), total litter weight^5^ (*P* = 0.077; coefficient = 5.13), alive litter weight^8^ (old vs. young, *P* = 0.002), NBW percentage^10^ (old vs. mid, *P* < 0.001; mid vs. young, *P* < 0.001), HBW percentage^11^ (old vs. young,* P* < 0.001; mid vs. young, *P* < 0.001), BW at d 26^14^ (old vs. mid, *P* < 0.001; old vs. young, *P* < 0.001), BW d 14 pw^16^ (old vs. young, *P* < 0.001; mid vs. young, *P* < 0.001)

Litter size was included as covariate for the pre-weaning performance and was significant for the BW of total born^3^ (*P* < 0.001; coefficient = 141.54), the CV of total born^4 ^(*P* < 0.001; coefficient = 61.59), total litter weight^5^ (*P* < 0.001; coefficient = 85.81), BW of born alive^6 ^(*P* < 0.001; coefficient = 130.37), CV of born alive^7^ (*P* < 0.001; coefficient = 46.63), alive litter weight^8^ (*P* < 0.001; coefficient = 22.22), LBW percentage^9^ (*P* < 0.001; coefficient = 1,080.70), NBW percentage^10^ (*P* < 0.001; coefficient = 25.93), HBW percentage^11^(*P* < 0.001; coefficient = 1144.37), BW d 6^12^ (*P* < 0.001; coefficient = 80.18), ADG d 0– 6^13^ (*P* = 0.027; coefficient = 4.90), ADG d 6–26^15^ (*P* = 0.077; coefficient = 3.12)

The interaction between the diet and the season was tested and resulted statistically significant for the BW at d 26 (*P* = 0.035) and tended to be statistically different for the ADG from d 0 to 14 pw (*P* = 0.07)

Means within a row with different superscripts differ for diets’ contrasts ^ABC ^*P* < 0.05 and ^abc^ *P* < 0.10; for seasons’ contrast ^YZ^ *P* < 0.05 and ^yz^*P* < 0.10

*BW*, Body weight; *CV*, Coefficient of variation; *LBW*, Low birthweight, BW < 1 kg; *NBW*, Normal birthweight, 1.0 kg ≤ BW ≤ 1.7 kg, *HBW*, High birthweight,  BW > 1.7kg; d 0, birth; d 26, weaning; d 14 pw, 14 days after weaning

^1^A total of 320 sows were divided into 4 experimental groups: CO, fed with a basal diet; Early35, fed with the CO diet with 21.8 g/d on-top Arg during the first 35 days of gestation; Late45, fed with the CO diet with 21.8 g/d on-top Arg during the last 45 days of gestation; COM, fed with the CO diet with 21.8 g/d on-top Arg during all gestation

^2^The trial was performed during 2 seasons: warm, sows performed gestation between July and September; cold, sows performed gestation between November and January

At birth, the BW and the CV of the total number of piglets born, the total weight of the litter, the BW and CV of the piglets born alive as well as the weight of the alive litter were not affected by diet. The percentage of LBW piglets was lower in the Early35 group as compared with the Late45 (*P* < 0.001), the COM (*P* < 0.001), and the CO (*P* = 0.020) group, and was lower in the CO group as compared with the Late45 (*P* = 0.050) and the COM (*P* < 0.001) groups. The percentage of HBW piglets was higher in the Late45 group as compared with the CO (*P* < 0.001), the Early35 (*P* = 0.042), and the COM (*P* < 0.001) groups, and was higher in the Early35 group as compared with the COM (*P* = 0.046) group. The percentage of NBW piglets was not influenced by diet.

On d 0, the BW of the total piglets born (*P* = 0.054), the CV at birth of the total number of piglets born (*P* = 0.087), and the total weight of the litter (*P* = 0.092) tended to be lower during the warm season. The BW of piglets born alive was lower during the warm season (*P* = 0.006). During the warm season, the weight of the alive litter tended to be lower than in the cold season (*P* = 0.063). Furthermore, the percentage of LBW piglets was higher during the warm season (*P* < 0.001) and the percentage of HBW piglets was lower during the warm season (*P* < 0.001), while no difference in the percentage of NBW piglets due to season was observed.

Bodyweight on d 6 and the ADG from d 0 to 6 and d 6 to 26, were not affected by diet or by season. Moreover, these variables showed no interaction.

Bodyweight at weaning (d 26) was influenced by the interaction between diet and season (*P* = 0.035); however, the contrasts did not show any differences among the groups. Diet did not affect BW at d 26 or the ADG from d 6 to 26 and from d 0 to 26, respectively. Season did not affect BW on d 26 or the ADG from d 6 to 26, while the ADG from d 0 to 26 was higher during the cold season (*P* = 0.006).

Bodyweight on d 14 post weaning (pw) and the ADG from d 26 to d 14 pw were not influenced by the interaction between diet and season, while the ADG from d 0 to d 14 pw tended to be influenced by the interaction between diet and season (*P* = 0.070); the Late45 group had a higher ADG from d 0 to d 14 pw compared with the CO group (*P* = 0.070) only during the warm season. In addition, BW on d 14 pw, the ADG from weaning to d 14 pw and the ADG from d 0 to d 14 pw (*P* < 0.001) were influenced by season, and were higher in the cold than the warm season.

**Fig. 1:**
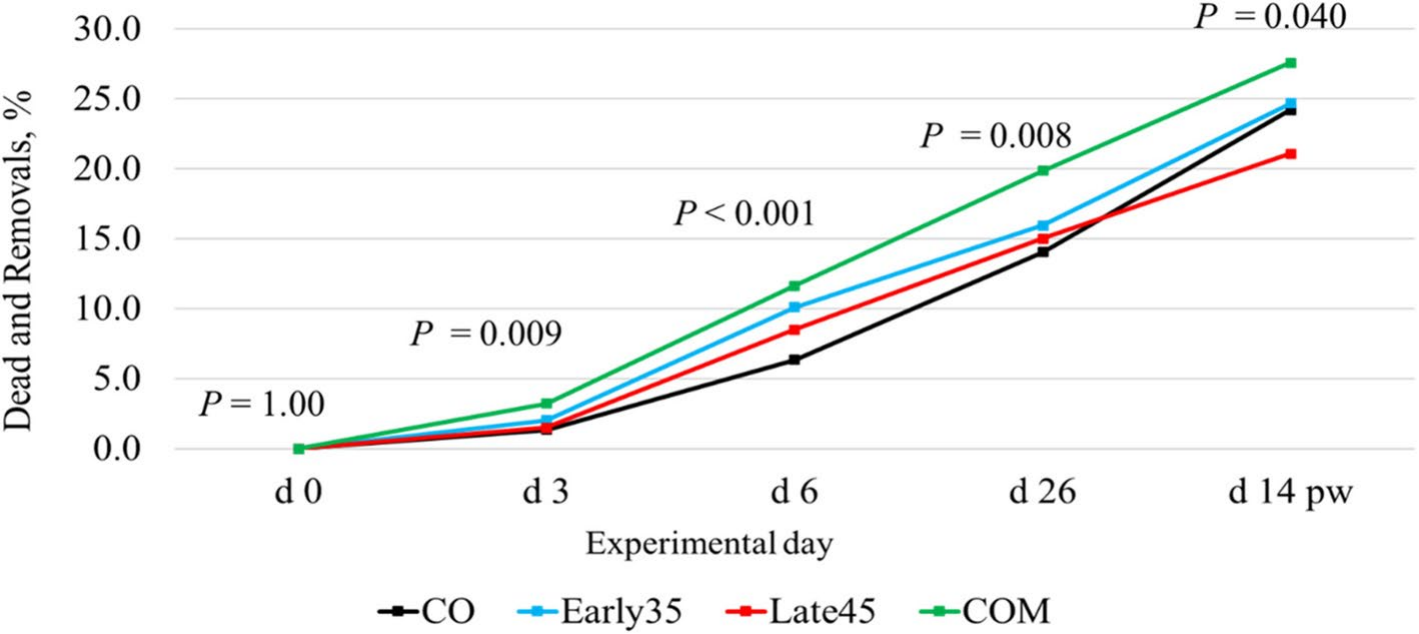
Effect of the diet on the mortality and exclusion rate of piglets during the study. A total of 320 sows were divided into 4 experimental groups: CO, fed with a basal diet; Early35, fed with the CO diet with 21.8 g/d on-top Arg during the first 35 d of gestation; Late45, fed with the CO diet with 21.8 g/d on-top Arg during the last 45 d of gestation; COM, fed with the CO diet with 21.8 g/d on-top Arg during all gestation. At d 3, mortality was higher in the COM group if compared to the CO (*P* = 0.020) and the Late45 group (*P* = 0.027). At d 6 the CO group had a lower mortality rate compared to the Early35 (*P* = 0.009) and to the COM group (*P* < 0.001), while tended to be lower in the Late45 group if compared to the COM group (*P* = 0.055). At d 26 COM had a higher mortality rate than the CO (*P* = 0.010) and the Late45 group (*P* = 0.033), while tended to be higher in COM group if compared to the Early35 (*P* = 0.070). At d 14 pw the COM group had a higher mortality rate than the Late45 group (*P* = 0.028)

The mortality and exclusion rates of piglets based on diet are reported in Fig. 1. On d 3, the mortality and exclusion rates were higher in the COM group as compared with the CO (*P* = 0.020) and the Late45 (*P* = 0.027) groups. On d 6, the mortality and exclusion rates were higher in the COM and Early35 groups as compared with the CO group (*P* < 0.001 and *P* = 0.009, respectively) and tended to be higher in the COM group as compared with the Late45 group (*P* = 0.055). At d 26, the COM group had higher mortality and exclusion rates than the CO (*P* = 0.010) and the Late45 (*P* = 0.033) groups and tended to be higher than the Early35 group (*P* = 0.070). On d 14 pw, the COM group had higher mortality and exclusion rates than the Late45 group (*P* = 0.028). Based on season, the mortality and exclusion rates were higher during the warm season on d 6 (*P* < 0.001), d 26 (*P* < 0.001) and d 14 pw (*P* < 0.001) (data not shown).

### Composition of colostrum

Table 5 reports the effect of diet and season on the proximal composition and Ig concentration of colostrum. The proximal composition of colostrum was not influenced by diet, season or by their interaction, except for the quantity of lactose which was higher during the cold season (*P* = 0.001). The concentration of IgM and IgA were not affected by diet, season or their interaction, while the concentration of IgG was higher in the CO group as compared with the other groups (*P* < 0.001), and it was higher during the warm season (*P* < 0.001).

**Table 5:** Effect of Arg supplementation and season on the proximal composition of sows’ colostrum

Items	Diet^1^					Season^2^			*P*-value	
	**CO**	**Early35**	**Late45**	**COM**	**SEM**	**Warm**	**Cold**	**SEM**	**Diet**	**Season**
Fat, g/100 mL	4.48	4.77	5.12	4.8	0.27	4.65	4.93	0.2	0.437	0.337
Protein, g/100 mL	16.7	16.8	16.7	15.9	0.38	16.4	16.7	0.28	0.304	0.337
Lactose, g/100 mL	2.64	2.6	2.68	2.82	0.1	2.51^Z^	2.86^Y^	0.07	0.368	0.001
Casein, g/100 mL	12.1	12.1	12.1	11.5	0.28	11.7	12.1	0.21	0.328	0.232
Urea, mg/100 mL	135	139	131	134	3.83	136	133	2.85	0.524	0.581
IgG, mg/mL	105.6^A^	58.0^C^	83.9^B^	79.0^B^	2.0	92.8^Y^	68.7^Z^	1.02	< 0.001	< 0.001
IgA, mg/mL	7.48	8.28	9.01	7.21	0.88	7.79	8.2	0.76	0.461	0.738
IgM, mg/mL	2.23	2.36	2.15	2.28	1.16	2.43	2.09	1.13	0.971	0.425

The interaction between the diet and the season was tested and removed from the statistical model if not statistically significant

Means within a row with different superscripts differ for diets’ contrasts ^ABC^ *P* < 0.05 and ^abc^ *P* < 0.10; for seasons’ contrast ^YZ^ *P* < 0.05 and ^yz^ *P* < 0.10

^1^ A total of 320 sows were divided into 4 experimental groups: CO, fed with a basal diet; Early35, fed with the CO diet with 21.8 g/d on-top Arg during the first 35 days of gestation; Late45, fed with the CO diet with 21.8 g/d on-top Arg during the last 45 days of gestation; COM, fed with the CO diet with 21.8 g/d on-top Arg during all gestation

^2^ The trial was performed during 2 seasons: warm, sows performed gestation between July and September; cold, sows performed gestation between November and January

### Metabolomic profile of colostrum

A total of 29 metabolites were identified in the sow colostrum.

**Fig. 2:**
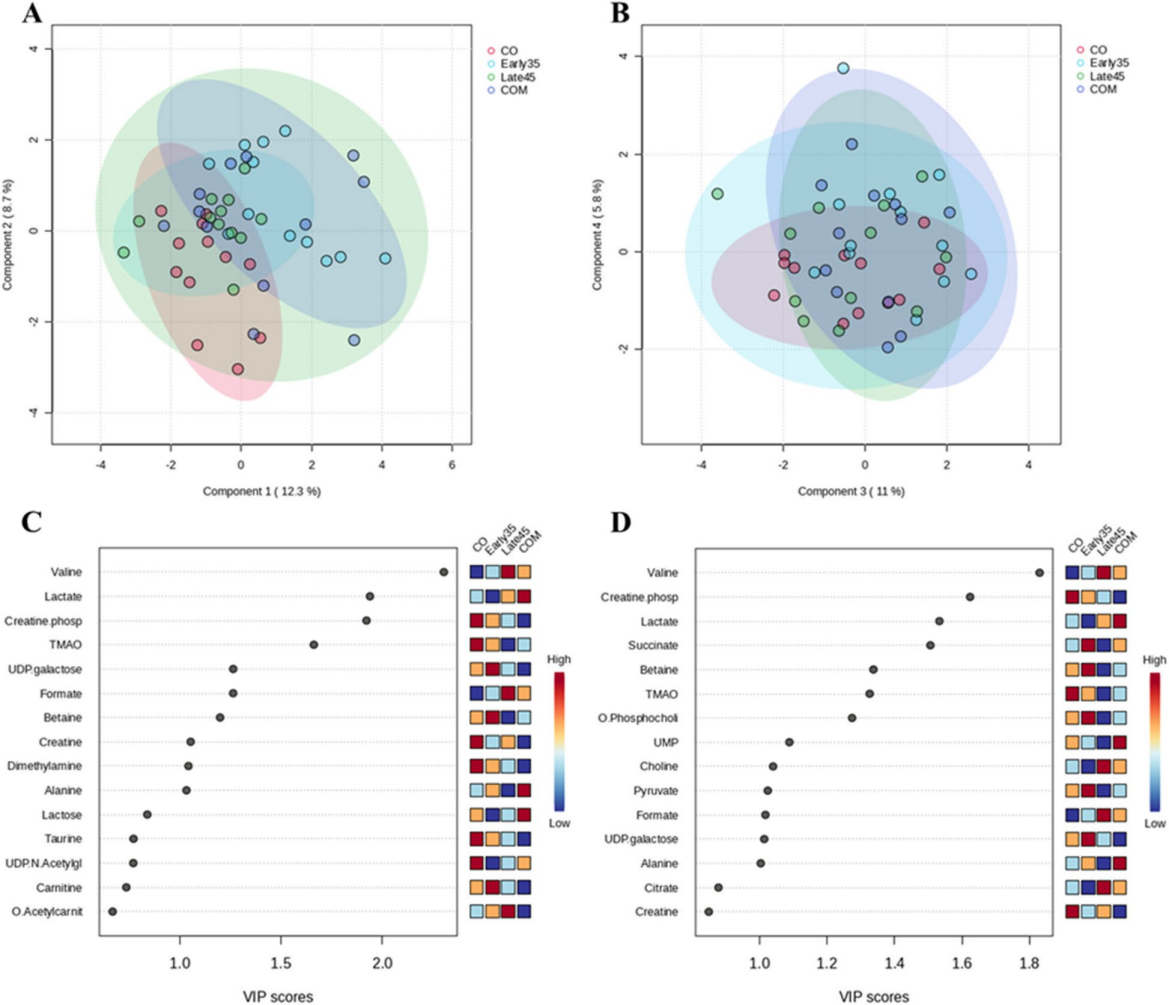
Effect of Arg supplementation on the metabolome characterization on the total colostrum spectra according to the experimental groups. A total of 320 sows were divided into 4 experimental groups: CO, fed with a basal diet; Early35, fed with the CO diet with 21.8 g/d on-top Arg during the first 35 d of gestation; Late45, fed with the CO diet with 21.8 g/d on-top Arg during the last 45 d of gestation; COM, fed with the CO diet with 21.8 g/d on-top Arg during all gestation. PLS-DA was applied to investigate differences in the metabolome profile. Score plots of the PC were reported. Although the overlapping of the experimental groups, a separation between COM and CO groups under the PC1 can be observed (**A**). The sources of variation for the most influential metabolites were displayed based on their VIP scores (**C** and **D**). The colored boxes on the right indicate the concentrations of the corresponding metabolite in each experimental group

A PLS-DA was applied to investigate the differences in the metabolome profile between the dietary groups and the seasons. Considering the dietary groups, the principal components 1 (PC1), 2 (PC2), 3 (PC3) and 4 (PC4) represented 12.3%, 8.7%, 11.0% and 5.8%, respectively of the colostrum spectra variance (Fig. 2A and B); although the four groups overlapped, a separation between the COM and the CO groups for PC1 could be observed. The sources of variation for the ten most influential metabolites among the dietary groups were displayed based on their VIP scores (VIP > 1.0) for PC1 (Fig. 2C) and PC2 (Fig. 2D). The CO group was characterized by a concentration of creatine phosphate notably higher (PC1: VIP score = 1.94; PC2: VIP score = 1.62) than the other groups, and by a notably higher concentration of Trimethylamine N-oxide (TMAO) (PC1: VIP score = 1.66; PC2: VIP score = 1.33) which was the lowest in the Late45 group. The Early35 group was characterized a notable concentration of Uridine diphosphate (UDP) galactose (PC1: VIP score = 1.26), especially when compared with the COM group, and by a high concentration of succinate (PC2: VIP score = 1.51), betaine (PC2: VIP score = 1.34) and O-phosphocholine (PC2: VIP score = 1.27), especially when compared with the Late45 group.

The Late45 group was characterized by a higher valine concentration (PC1: VIP score = 2.30; PC2: VIP score = 1.83), especially when compared with the Early35 group. The COM group was characterized by a high lactate concentration (PC1: VIP score = 1.92; PC2: VIP score = 1.53), especially when compared with the Early35 group.

Additional file 1: Table S1 reports the effect of diet and season on the metabolites identified in the colostrum samples.

The CO group had more uridine than the Early35 group (*P* < 0.001), more UDP galactose than the Late45 (*P* < 0.001) and the COM (*P* < 0.001) groups, more UDP glucuronate than the Early35 group (*P* < 0.001), more UDP- n-acetylglucosamine (*P* < 0.001) than all the other groups, more sn-glycero-3-P-choline than the COM group (*P* < 0.001), more citrate than the Early35 (*P* < 0.001) and the COM (*P* < 0.001) groups, more succinate and more pyruvate than the Late45 group (*P* < 0.001), and more O-Acetylcarnitine than COM group (*P* < 0.001).

The Early35 group had the highest concentration of UDP galactose (*P* < 0.001); it tended to have more UDP glucose than the COM group (*P* = 0.060), more sn-glycero-3-P-choline than all the other groups (*P* < 0.001), more succinate than the CO (*P* < 0.001) and the Late45 groups (*P* < 0.001), more pyruvate than all the other groups (*P* < 0.001), more O-Acetylcarnitine than the CO (*P* < 0.001) and the COM groups (*P* < 0.001), and more acetate than the CO group (*P* < 0.001).

The Late45 group had the highest concentrations of formate (*P* < 0.001) and of uridine (*P* < 0.001), more UDP galactose than the COM group (*P* < 0.001), more UDP glucuronate than the CO (*P* = 0.005) and the Early35 groups (*P* < 0.001), more UDP-n-acetylglucosamine than the Early35 group (*P* < 0.001), more sn-glycero-3-P-choline than the CO (*P* < 0.001) and the COM (*P* < 0.001) groups, more choline than the Early35 group (*P* = 0.005), the highest concentrations of citrate (*P* < 0.001) and O-acetylcarnitine (*P* < 0.001), more acetate than the CO (*P* < 0.001) and the COM groups (*P* = 0.019), and more valine than the CO (*P* = 0.001) and the Early35 (*P* = 0.002) groups.

The COM group had more formate than the CO (*P* = 0.002) and the Early35 groups (*P* = 0.003), more uridine than the Early35 group (*P* < 0.001), more UDP than the CO and the Early35 groups (*P* < 0.001), more UDP- n-acetylglucosamine than the Early35 group (*P* < 0.001), more citrate than the Early35 group (*P* < 0.001), the highest concentrations of succinate (*P* = 0.010), more pyruvate than the Late45 group (*P* < 0.001), more acetate than the CO group (*P* = 0.001), the highest concentrations of alanine (*P* = 0.005) and lactate (*P* < 0.001), and more valine than the CO and the Early35 groups (*P* < 0.001).

The colostrum metabolic profile was also influenced by season as shown using a PLS-DA model and ANOVA analysis in Fig. S1 and Table S1. Briefly, according to the PLS-DA analysis, the 2 seasons were separated along the PC2 with the warm season being characterized by a higher concertation of uridine (PC1: VIP score = 2.35; PC2: VIP score = 2.10), UDP-glucuronate (PC1: VIP score = 1.35), O-phosphocholine (PC1: VIP score = 1.27) and sn-glycero-3-phosphocholine (PC2: VIP score = 1.35). Conversely, O-acetylcarnitine (PC1: VIP score = 1.93; PC2: VIP score = 1.62) and succinate (PC1: VIP score = 1.51; PC2: VIP score = 1.27) were higher during the cold season. According to the ANOVA, in addition to the above-mentioned metabolites, the warm season had a higher concentration of citrate (*P* < 0.001) and lactate (*P* = 0.025), and more UDP galactose (*P* < 0.001), UDP glucose (*P* = 0.018), myo-Inositol (*P* = 0.027), pyruvate (*P* < 0.001), O-acetylcarnitine (*P* < 0.001), and acetate (*P* < 0.001) than in the cold season.

### Metabolomic profile of sow urine

A total of 61 metabolites were identified in the sow urine on d 35 of gestation.

**Fig. 3:**
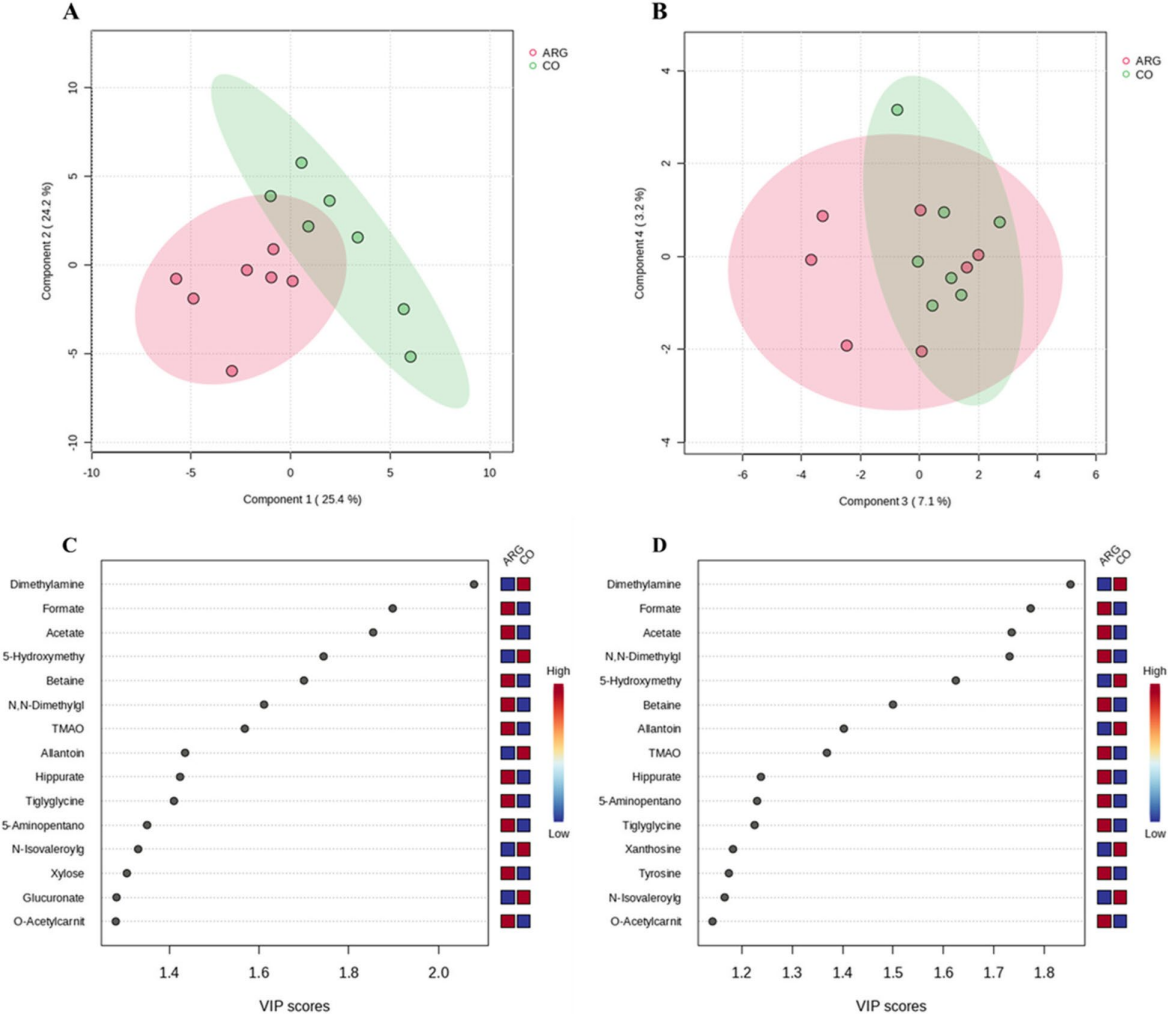
Effect of Arg supplementation on the metabolome characterization on the total urine spectra at d 35 of gestation, according to the experimental groups. Scores plot of PLS-DA between components 1 and 2 (**A**) and components 3 and 4 (**B**) on the total urine spectra based on the experimental groups: CO (CO + Late45) vs. ARG (Early35 + COM). Important features identified by PLS-DA in components 1 (**C**) and 2 (**D**), respectively. The colored boxes on the right indicate the relative concentrations of the corresponding metabolite in each group under study: CO (CO + Late45) vs. ARG (Early35 + COM)

A PLS-DA was applied to investigate the differences in the metabolome composition of the urine between CO (CO + Late45) and ARG (Early35 + COM) groups. The PC1, PC2, PC3 and PC4 represented 25.4%, 24.2%, 7.1% and 3.2%, respectively of the colostrum spectra variance (Fig. 3A and B). Although the groups partially overlapped, a separation could be observed between them along PC1. The metabolites which, for the most part, determined this separation were searched for by calculating their VIP scores (VIP > 1.0) for PC1 (Fig. 3C) and PC2 (Fig. 3D). The CO group had higher scores for dimethylamine (PC1: VIP score = 2.08; PC2: VIP score = 1.85), 5-hydroxymethyl-4-methyluracil (PC1: VIP score = 1.74; PC2: VIP score = 1.62), and allantoin (PC1: VIP score = 1.44; PC2: VIP score = 1.40), while the ARG group had higher scores for formate (PC1: VIP score = 1.90; PC2: VIP score = 1.77), acetate (PC1: VIP score = 1.85; PC2: VIP score = 1.74), betaine (PC1: VIP score = 1.70; PC2: VIP score = 1.50), N,N-Dimethylglycine (PC1: VIP score = 1.61; PC2: VIP score = 1.73), TMAO (PC1: VIP score = 1.57; PC2: VIP score = 1.37), hippurate (PC1: VIP score = 1.42), and tiglyglycine (PC1: VIP score = 1.41).

Table S2 reports the identification of the metabolites, their relative concentrations in urine samples on d 35 of gestation and the effect of the Arg supplementation according to the ANOVA analysis. When compared with the ARG group, the CO group had higher concentrations of 4-hydroxyphenylacetate (*P* = 0.002), *cis*-aconitate (*P* < 0.001), allantoin (*P* < 0.001), glucuronate (*P* < 0.001), arabinose (*P* = 0.031), ascorbate (*P* < 0.001), gluconate (*P* < 0.001), glycine (*P* < 0.001), creatine (*P* < 0.001), 5-hydroxymethyl-4-methyluracil (*P* = 0.043), N-acetylglucosamine (*P* = 0.040), N-acetylglutamate (*P* = 0.017), lysine (*P* < 0.001), lactate (*P* < 0.001), 2,3-butanediol (*P* = 0.048), leucine (*P* < 0.001), and N-isovalerylglycine (*P* = 0.029). Conversely, the ARG groups had higher concentrations of formate (*P* = 0.010), isobutyrate, (*P* < 0.001), tyrosine (*P* < 0.001), cytosine (*P* < 0.001), glucose (*P* < 0.001), xylose (*P* < 0.001), betaine (*P* < 0.001), mannitol (*P* = 0.009), TMAO (*P* < 0.001), N,N-dimethylglycine (*P* < 0.001), 5-aminopentanoate (*P* = 0.001) and acetate (*P* < 0.001).

### Metabolomic profile of the sow feces

A total of 59 metabolites were identified in the sow feces on d 35 of gestation.

**Fig. 4:**
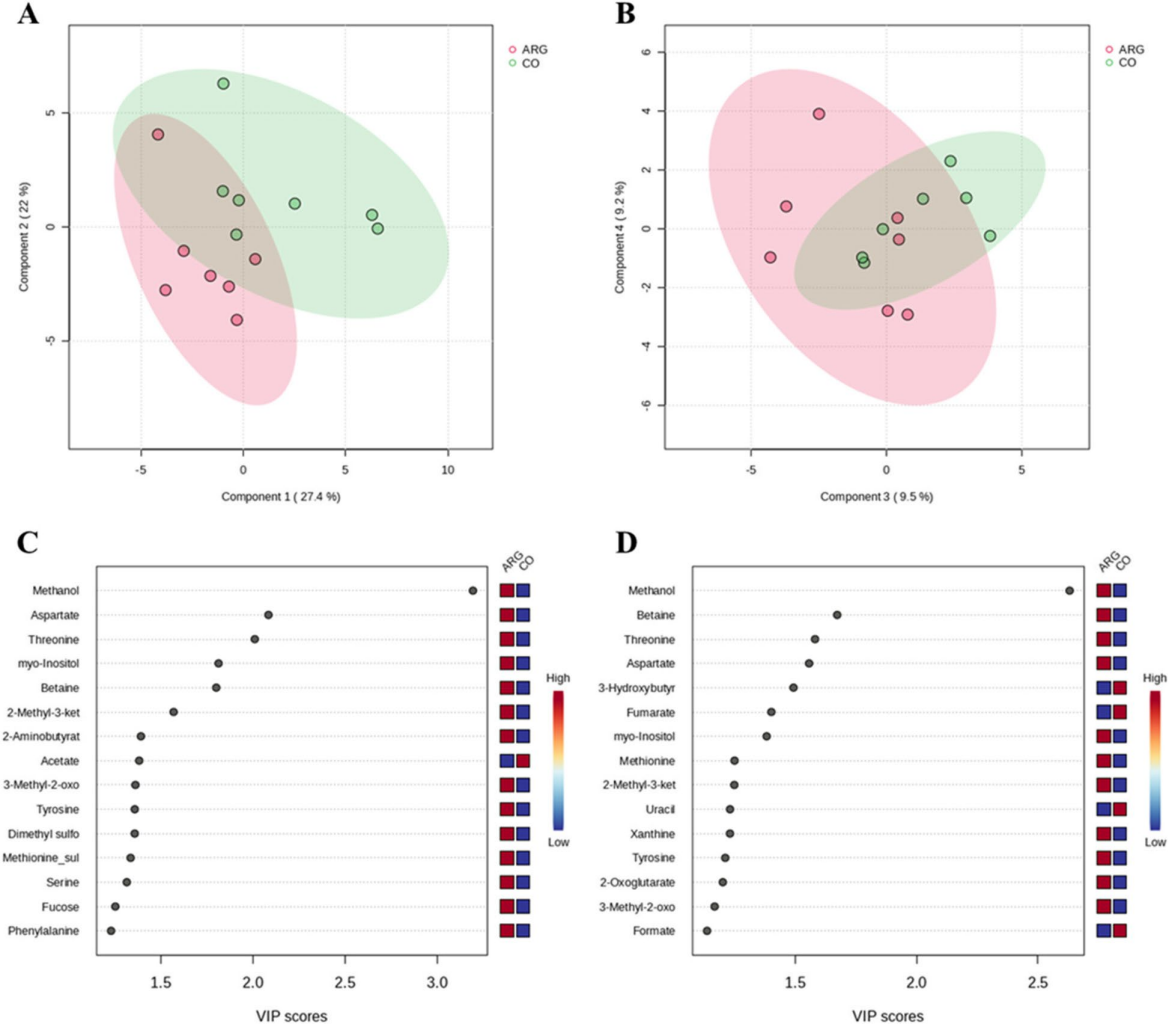
Effect of the Arg supplementation on the metabolome characterization on the total fecal spectra at d 35 of gestation, according to the experimental groups. Scores plot of PLS-DA between components 1 and 2 (**A**) and components 3 and 4 (**B**) on the total fecal spectra based on the experimental groups: CO (CO + Late45) vs. ARG (Early35 + COM). Important features identified by PLS-DA in principal components 1 (**C**) and 2 (**D**), respectively. The colored boxes on the right indicate the relative concentrations of the corresponding metabolite in each group under study: CO (CO + Late45) vs. ARG (Early35 + COM)

The PLS-DA was applied to investigate the differences in the metabolome composition of the fecal samples between the CO (CO + Late45) and the ARG (Early35 + COM) groups. The PC1, PC2, PC3 and PC4 of the model represented 27.4%, 22.0%, 9.5%, and 9.2%, respectively (Fig. 4A and B). Although the groups partially overlapped, a separation could be observed between them along PC1 (Fig. 4A). The metabolites which, for the most part, led to this separation were searched for by calculating their VIP scores (VIP > 1.5) for PC1 (Fig. 4C) and PC2 (Fig. 4D). The CO group was characterized by higher scores for acetate (PC1: VIP score = 1.38), 3-hydroxybutyrate (PC2: VIP score = 1.49), and fumarate (PC2: VIP score = 1.40). The ARG groups showed higher scores for methanol (PC1: VIP score = 3.19; PC2: VIP score = 2.63), aspartate (PC1: VIP score = 2.08; PC2: VIP score = 1.56), threonine (PC1: VIP score = 2.01; PC2: VIP score = 1.58), myo-inositol (PC1: VIP score = 1.81; PC2: VIP score = 1.38), betaine (PC1:VIP score = 1.80; PC2: VIP score = 1.67) and 2-methyl-3-ketovalerate (PC1: VIP score = 1.57).

Table S3 reports the metabolites identified, their relative concentrations, and the effect of diet and season on the fecal samples on d 35 of gestation. The CO group had higher concentrations of uracil (*P* = 0.004) and galactose (*P* = 0.028), while the ARG group had more methanol (*P* = 0.025) and tended to have more 2-Oxoisocaproate (*P* = 0.071).

A total of 61 metabolites were identified in the sow fecal samples collected on d 106 of gestation. The PLS-DA analysis did not detect specific clusters linked to diet (Fig. S2); similarly, the ANOVA analysis did not evidence differences in the concentrations of the metabolites owing to the diet (Table S4).

However, differences linked to season were observed. According to the season, the PC1, PC2, PC3, and PC4 represented 43.9%, 12%, 5.7% and 5% of the fecal spectra variance, respectively (Fig. S3). A separation between them along PC1 and PC2 could be observed. In the warm season, isobutyrate (PC1: VIP score = 1.79; PC2: VIP score = 1.45), isovalerate (PC1: VIP score = 1.68), levulinate (PC1: VIP score = 1.36; PC2: VIP score = 1.87), acetoin (PC1: VIP score = 1.36; PC2: VIP score = 1.98), 3-yydroxybutyrate (PC2: VIP score = 1.59) and acetoacetate (PC2: VIP score = 1.39) were higher than in the cold season. The samples collected in the cold season scored higher for thymine (PC1: VIP score = 2.33; PC2: VIP score = 1.58), hypoxanthine (PC1: VIP score = 1.99), uracil (PC1: VIP score = 1.92), fumarate (PC1: VIP score = 1.91), betain (PC1: VIP score = 1.81), myo-inositol (PC1: VIP score = 1.55), glutamate (PC1: VIP score = 1.45) and lactate (PC1: VIP score = 1.38).

Considering the ANOVA models, the samples collected in the warm season showed higher concentrations of phenylacetate (*P* = 0.005), threonine (*P* = 0.029), isobutyrate (*P* = 0.002), acetoacetate (*P* = 0.017), acetoin (*P* = 0.007), thymine (*P* < 0.001), cadaverine (*P* = 0.022) and isovalerate (*P* = 0.013), and a tendency towards higher concentrations of 3-hydroxyphenylacetate (*P* = 0.071), valerate (*P* = 0.078), fucose (*P* = 0.099) and 3-hydroxybutyrate (*P* = 0.057). During the cold season, higher concentrations were observed for hypoxanthine (*P* = 0.007), fumarate (*P* < 0.001), uracil (*P* = 0.014), lactate (*P* = 0.040) and betaine (*P* = 0.003), while a tendency towards higher concentrations was observed for myo-inositol (*P* = 0.071) and glutamate (*P* = 0.078).

### Fecal microbiota of sows

**Fig. 5:**
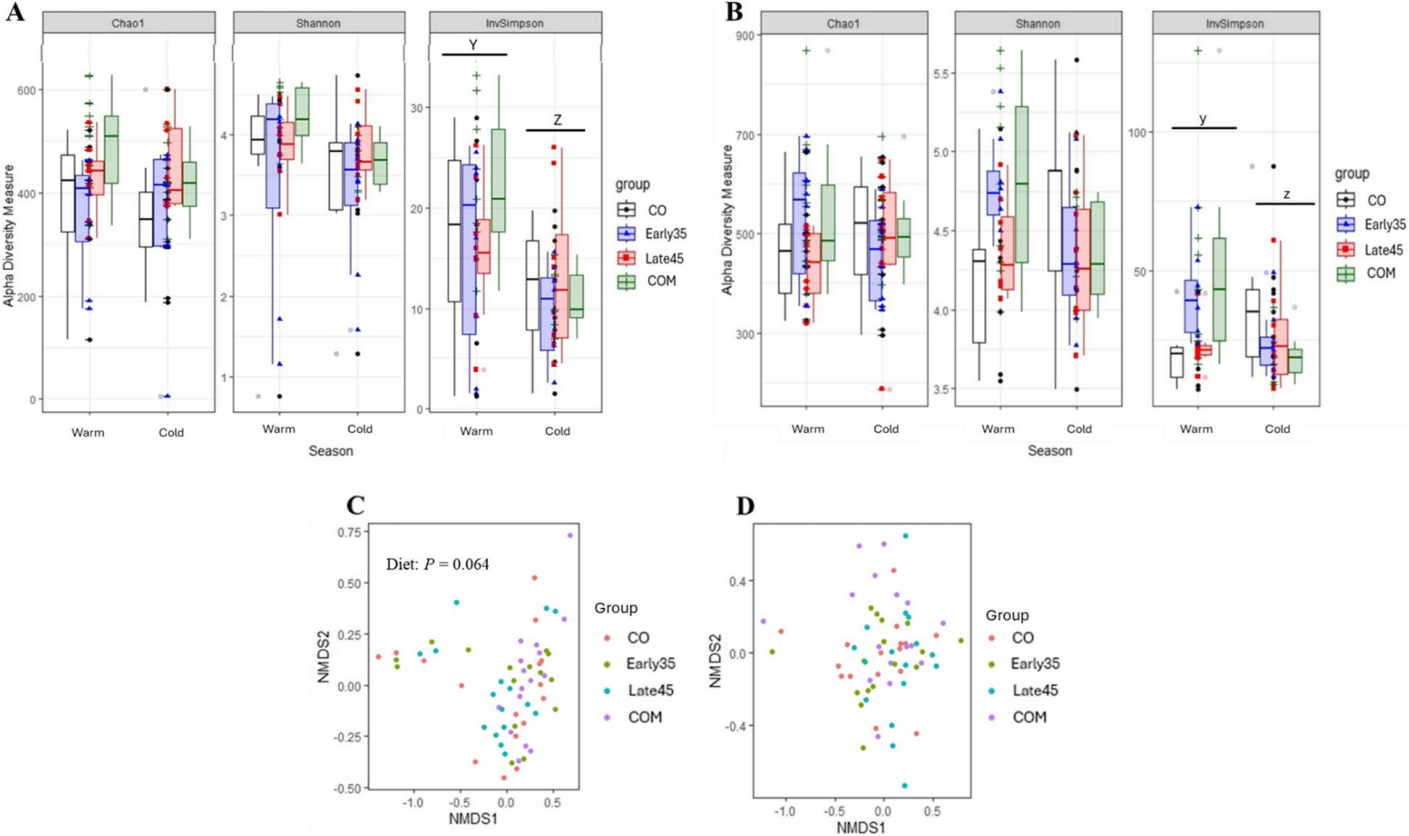
Effect of Arg supplementation on alpha and beta diversity indices of sows feces at d 35 and d 106 of gestation. No differences were observed between the groups at d 35 of gestation (**A**). InvSimpson index at d 35 (**A**) was higher during the warm season if compared to the cold season (*P* = 0.001), while at d 106 (**B**) tended to be higher during the warm season if compared to the cold one (*P* = 0.085). Beta diversity tended to be affected by the diet at d 35 (**C**, R^2^ = 0.069; *P* = 0.064), but not at d 106 (**D**, R^2^ = 0.041). Different superscripts differ for diets’ contrasts ^AB^ *P* < 0.05 and ^ab^ *P* < 0.10; for seasons’ contrast ^YZ^ *P* < 0.05 and ^yz^ *P* < 0.10

Figure 5 reports the effects of diet and season on the alpha and beta diversity indices of the fecal samples collected on d 35 and d 106. Diet, and the interaction between diet and season did not affect the Chao1, Shannon, and InvSimpson indices at any timepoint. On d 35, the InvSimpson index was higher during the warm season (*P* = 0.001) while on d 106, the InvSimpson index tended to be higher during the warm season (*P* = 0.085).

The interaction between diet and season did not affect the beta diversity. The beta diversity tended to be affected by diet (Fig. 5A, R^2^ = 0.069; *P* = 0.064) on d 35 and was not affected by diet on d 106 (Fig. 5B). Season influenced the beta diversity on d 35 (Fig. 5C, R^2^ = 0.061; *P* = 0.002) and d 106 (Fig. 5D, R^2^ = 0.104; *P* < 0.001).

No difference in the taxa abundancy due to diet was found by the LefSE analyses on d 35 and d 106, while a total of 24 taxa on d 35 (Table S5) and 83 taxa on d 106 (Table S6) were influenced by season.

### Short chain fatty acid and ammonia concentrations in sow feces

Table 6 reports the effects of diet and season on the SCFA and ammonia concentrations in the sow feces on d 106 of gestation. The interaction between diet and season did not affect the SCFA and the ammonia concentrations.

**Table 6:** Effect of Arg supplementation and season on the concentration of SCFA and ammonia in sows’ feces at d 106 of gestation

Items	Diet^1^					Season^2^			*P*-value	
	**CO**	**Early35**	**Late45**	**COM**	**SEM**	**Warm**	**Cold**	**SEM**	**Diet**	**Season**
Lactic ac., µmol/g	11.38^AB^	9.59^AB^	16.50^A^	11.57^B^	0.83	10.7^Z^	13.5^Y^	0.63	< 0.001	0.001
Acetic ac., µmol/g	264^A^	246^B^	248^B^	245^B^	4.00	239^Z^	263^Y^	2.91	0.002	< 0.001
Propionic ac., µmol/g	68.2	78.3	72.9	97.5	10.1	64.7^Z^	93.8^Y^	7.26	0.186	0.005
Isobutyric ac., µmol/g	60.9^B^	121.4^A^	63.2^B^	57.2^B^	2.30	62.7^Z^	82.5^Y^	1.54	< 0.001	< 0.001
Butyric ac., µmol/g	116.5^A^	109.2^A^	81.0^C^	95.7^B^	2.62	106.3^Y^	93.4^Z^	1.84	< 0.001	< 0.001
Isovaleric ac., µmol/g	12.2	10.0	10.7	13.5	1.28	13.0^Y^	10.2^Z^	0.92	0.209	0.032
Valeric ac., µmol/g	7.94^A^	3.09^B^	6.46^A^	3.67^B^	0.63	5.70^Y^	4.23^Z^	0.41	< 0.001	0.008
Ammonia, µmol/g	20.0^AB^	17.6^B^	18.5^B^	23.5^A^	1.13	2.93^z^	3.04^y^	0.041	< 0.001	0.079

The interaction between the diet and the season was tested and removed from the statistical model if not statistically significant

Means within a row with different superscripts differ for diets’ contrasts ^ABC^ *P* < 0.05 and ^abc^ *P* < 0.10; for seasons’ contrast ^YZ^ *P* < 0.05 and ^yz^ *P* < 0.10

^1^ A total of 320 sows were divided into 4 experimental groups: CO, fed with a basal diet; Early35, fed with the CO diet with 21.8 g/d on-top Arg during the first 35 d of gestation; Late45, fed with the CO diet with 21.8 g/d on-top Arg during the last 45 d of gestation; COM, fed with the CO diet with 21.8 g/d on-top Arg during all gestation

^2^ The trial was performed during 2 seasons: warm, sows performed gestation between July and September; cold, sows performed gestation between November and January

The CO group had a concentration of acetic acid higher than all the other groups (*P* = 0.020); it had more butyric acid than the Late45 and the COM groups (*P* < 0.001), had more valeric acid than the Early35 and the COM groups (*P* < 0.001). The Early35 group had more isobutyric acid than all the other groups (*P* < 0.001), and more butyric acid than the Late45 (*P* < 0.001) and the COM (*P* = 0.001) groups. The Late45 group had more lactic acid than the COM group (*P* < 0.001), and more valeric acid than the Early35 (*P* < 0.001) and the COM groups (*P* = 0.002). The COM group had more ammonia than the Early35 (*P* = 0.001) and the Late45 (*P* = 0.012) groups, while it had more butyric acid than the Late45 group (*P* < 0.001).

The samples collected during the warm season had more butyric (*P* < 0.001), isovaleric (*P* = 0.032) and valeric acids (*P* = 0.008). During the cold season, the concentration of ammonia tended to be higher (*P* = 0.079), while higher concentrations of lactic (*P* = 0.001), acetic (*P* < 0.001), propionic (*P* = 0.005) and isobutyric acids (*P* < 0.001) were observed.

## Discussion

This study highlighted the different effects of the on-top addition of Arg on sow reproductive performance. The interaction between diet and season was never significant, except for a few tendencies regarding litter growth. For the most part, this result led to discussing the outcomes obtained from the supplementation of Arg irrespective of season, suggesting that the dose of Arg used was not capable of alleviating heat stress in sows. In terms of productive performance, the results suggested differing effects of Arg, depending on the period of supplementation.

The positive effect of Arg supplementation in early gestation is widely stated in the literature [41, 42]. This study confirmed the potential interest in and the pivotal role of Arg at the initial stage of gestation. In fact, although the total number of piglets born and born alive did not increase in the Early35 group, the percentage of stillborn and mummified piglets was reduced as compared with the unsupplemented group. Furthermore, the Early35 group had a lower percentage of LBW piglets as compared with all the other groups. Since the literature reports a higher rate of fetal mortality during d 12–15 and on d 30 of gestation [43], these positive effects could be attributed to the improvement in the placental angiogenesis and fetal implantation obtained via the use of additional crystalline Arg [17] in the first 35 d of gestation. The lack of any effect on the part of the Early35 group in improving the number of total number of piglets born, born alive and their BW is in agreement with the findings of Li et al. [42] whose study found that a total dose of 48.61 g/d of Arg during the first 30 d of gestation did not affect the litter size and the BW at birth. Although Arg supplementation in the first 35 d did not lead to an increase in piglet BW, it did reduce the percentage of LBW piglets which is an emerging problem for highly-prolific sows, since they are weaker than NBW piglets and have a higher mortality rate or a slower growth rate during the suckling and the post-weaning periods [27].

On the other hand, the use of Arg in the last 45 d of gestation reduced the number of stillborn piglets and increased the percentage of HBW piglets in agreement with the findings of Nuntapaitoon et al. [19] who gave a total dose of 46.75 g/d of Arg during late gestation (from d 85 until farrowing). These effects can be attributed to the role of Arg in regulating placental-fetal blood flow by improving nutrients and oxygen transfer to the fetuses via the umbilical cord [44]. Placental vascularization reaches near peak by d 70 of gestation [45], and fetuses grow rapidly, with approximately 60% of their growth occurring during the final third of gestation [46]. Therefore, the increase in the placental-fetal blood flow due to Arg supplementation in this latter period could have contributed to better growth of the fetuses. Moreover, during late gestation, the development of fetus muscle is mainly characterized by hypertrophy [47]. This process is governed by the mTOR pathway and is facilitated by insulin-like growth factor 1. As stated by Takahara et al. [48], Arg is known to potently activate the mTOR pathway. Nevertheless, the present results suggested that the improvement in nutrients and oxygen transfer derived from Arg was not equally distributed across the fetuses since no general increase in birth weight was observed but rather an increase in the percentage of pigs with an HBW was observed. However, the total daily dose of 38.3 g/d of Arg during late gestation did not affect piglet BW at birth. The lack of a general increase in piglet BW at birth in the Late45 group was in contrast with previous studies by Hong et al. [21] and Wu et al. [49], who administered a total dose of 39.56 g/d and 33.4 g/d of Arg, respectively to the sows. However, it agreed with the study of Bass et al. [50] who administered a total of 42.5 g/d of Arg. These discrepancies in terms of Arg effects could be due to the composition of the basal diet, the management of the farm or the genetics of sows involved in the trials. The anticipated effects of Arginine supplementation on pregnant sows, such as increased litter size and birth weight, were based on studies such as those by Gao et al. [51]. In their research, the total dose of Arg was 34.2 g/d from days 22 to 89 of gestation and 51.3 g/d from d 90 to farrowing. Similarly, da Silva et al. [52] provided a total dose of 43.7 g/d of Arg between d 30–60 and 80–114 of gestation. The present results were not in agreement with these previous studies. In fact, the group which received the additional Arg during the entire period of gestation did not show an increase in the total number of piglets born and piglets born alive or an increase in the birth body weight of the piglets. Moreover, an increase in the percentages of LBW and crushed piglets was observed; LBW piglets are weaker than NBW piglets and have inadequate thermoregulation capability [53]. For this reason, they are more likely to stay close to the sow and consequently to be crushed, increasing pre-weaning mortality [54, 55]. In addition, the COM group had a numerically higher number of piglets alive at birth which could be partially related to the higher percentage of crushed piglets. Furthermore, this numeric increase in alive piglets could partially justify the increase in the sow ADG and BW observed in the COM group on d 106. This result contrasted with the findings of Mateo et al. [56] in which a total dose of 29.4 g/d of Arg fed to sows showed no effect on their BW. However, the dose in the study by Mateo et al. [56] was lower than the total 38.3 g/d dose used in the present study.

Except for a higher ADG from birth to 14 d after weaning in the Late45 group, only during the warm season, the growth performance of the piglets was not affected by diet. Similar to the ADG, sows’ diet did not have any positive effect on mortality and culling. These results were consistent with the meta-analysis the Authors published last year regarding the effects of Arg supplementation on gestating sows [15]. Arginine is rapidly metabolized in several pathways [57] which could explain why it is difficult to observe a residual effect during lactation when supplementation occurs only during the gestation period. In addition, when considering the lack of a significant effect of Arg supplementation on the proximal composition of colostrum, no difference in growth performance or mortality of piglets was expected during the suckling period. The lack of a significant effect of Arg on the sows’ colostrum composition agreed with the finding of Hong et al. [21] (39.56 g/d of total Arg). Unexpectedly, in the present study, Arg supplementation led to a reduction in the colostral concentration of IgG, contrary to the observations of Nuntapaitoon et al. [19]. Even though the colostrum yield was not assessed, this reduction was likely a consequence of an increase in colostrum production [58].

Although Arg supplementation had a limited impact on the colostrum composition, the metabolomic analysis of this matrix revealed important effects. Furthermore, the application of the metabolomic technique to the other matrices analyzed, such as feces and urine, allowed studying the effects which Arg supplementation may have on the physiology and metabolism of sows as well the possible reverberation on their litters in greater depth. Arginine plays an important role in the regulation of the nutrient metabolism; however, the mechanisms have not yet been clearly understood. An effect of Arg on the creatine and the nitric oxide (NO) pathways was observed in both colostrum and urine; the CO group was characterized by a higher concentration of creatine and creatinine phosphate in the colostrum. Arginine is converted to creatine in several steps; however, the first reaction is controlled by the enzyme L-arginine:glycine amidinotransferase [59]. To initiate the reaction, this enzyme requires adequate levels of glycine and minimal concentrations of creatine which can have a negative feedback on this pathway, thereby facilitating the use of arginine in the NO pathway [59]. Therefore, the metabolic results in the colostrum of the Late45 and the COM groups suggested a limitation in the use of Arg in the pathway for creatinine production, leading to a greater use of Arg in the NO production pathway. This pathway may also have been favored by the imbalance between the amount of Arg and glycine in the Arg supplemented groups. Glycine was not detected in the colostrum; however, a higher concentration of glycine and creatine was observed in urine on d 35 in the CO group as compared with the ARG group. Furthermore, according to Xu et al. [60], a high concentration of milk glycine, creatine phosphate, TMAO, and UDP-galactose, along with other metabolites, are associated with a negative energy balance in lactating cows. Interestingly, these metabolites were found in lower concentrations in the colostrum of the Late45 and the COM groups, suggesting a better energy status of these groups. On the contrary, these 2 groups had a higher concentration of colostrum valine and lactate, both associated with a good energy balance [60]. Furthermore, this result is consistent with the higher BW and ADG of the sows receiving Arg throughout gestation.

In addition, the Late45 group was characterized by a higher concentration of choline and a lower concentration of betaine; as described by [61], choline and betaine pathways are closely related since betaine can be produced by animals from choline by means of a two-step oxidation via the intermediate, betaine aldehyde, and the involvement of two enzymes (choline dehydrogenase and betaine aldehyde dehydrogenase). However, betaine can also be produced by microorganisms, both via betaine aldehyde and by a three-step methylation of glycine [61]. The low availability of glycine due to the Arg-to-creatine pathway may have reduced the production of betaine; in fact, as observed by Meadows and Wargo [62], the low concentration of betaine and TMAO is a consequence of the low concentration of carnitine observed in the colostrum. Moreover, the supplementation of Arg during late gestation significantly altered the metabolic pathways within TCA cycle. Indeed, while the concentration of pyruvate was reduced, the metabolic flux was redirected towards the production of acetate, leading to a subsequent increase in citrate concentration [63]. This shift in metabolic focus underscores the crucial role of Arg in modulating energy metabolism. Interestingly, the supplementation of Arg throughout the entire gestation altered the normal TCA cycle, promoting the production of alanine, lactate, and valine. The production of these metabolites instead of the classic TCA cycle intermediates is not entirely clear to the authors. In fact, the same metabolic pathway was observed by Hasegawa et al. [64]. Nonetheless, we believe that further investigation is necessary to deepen our understanding of the effects of Arg supplementation during sows’ gestation and its impact on metabolome. In addition to the effect of Arg on the carnitine pathway, a change in the protein metabolism involving intestinal microbial activity was observed in the urine. Arginine supplementation increased the concentrations of formate, acetate, TMAO, hippurate, betaine, and N,N-dimethylglycine in the urine. These metabolites were previously associated with intestinal microbial activity and abundance. Hippurate is derived from the microbial degradation of flavonol products [65]; TMAO is derived from the renal and hepatic oxidation of TMA which, in turn, can be derived from the microbial conversion of choline, carnitine, or betaine [66]. Formate and acetate are known end-products of various bacterial families deriving from the glucose and the AA metabolisms [67]. The bacteria present in the lumen of the small intestine also convert some dietary Arg into ornithine, proline, and polyamines (putrescine, spermine and spermidine) [68]. Therefore, the gastrointestinal microbiota can influence both the availability of Arg and the health of the host [69]. For example, in post-weaning pigs the intestinal catabolism of Arg decreases the amount of dietary Arg that enters in the portal vein [69]. According to the study conducted by Wu et al. [70], about 40% of the Arg is involved in catabolism and protein synthesis by the small intestine in adult pigs.

However, although these results suggest a strong involvement of Arg in the microbiome metabolism, only a slight trend in the beta diversity index was observed in the microbiome profile on d 35. Furthermore, no difference was observed in the microbiome profile and the abundance of specific microbiome taxa on d 106 of gestation. Previous studies had shown a slight effect of Arg on the fecal microbial profile of sows during gestation and lactation; however, even in these studies, the effect on the microbial composition was mild [8, 25]. To explain this effect, one can consider that the microbial profile of sows is considered to be stable as compared with that of younger animals.

Consistent with the urinary metabolomic profile, the fecal metabolomic profile, especially on d 35 of gestation, was influenced by Arg administration. Specifically, an increase in fecal AAs, especially aspartate, threonine, tyrosine, serine, and phenylalanine, was observed in the Arg group. This increase in different fecal AAs could be related to the increased availability of Arg-derived NO and its effect on some microorganisms. In fact, NO is known to alter the metabolism of bacteria [71]. The reduction in microbial activity could therefore have reduced AA catabolism in the intestinal lumen, justifying the higher fecal AA concentration as suggested by He et al. [72]. This effect on AAs, clearly observed on d 35, did not maintain its clarity until d 106. This apparently controversial result could have been due to the ability of the intestinal microbiota to shape its metabolism and adapt to the diet according to its evolutionary capacity [73]. Alternatively, it is equally possible that the different physiological situations of the sow between d 35 and 106 of gestation could have contributed to the diversity observed in the fecal metabolome at the 2 timepoints. In addition, although the fecal AA profile was not affected, the group receiving the Arg during the entire gestation period had a higher fecal ammonia concentration on d 106, suggesting an increase in nitrogen loss due to high Arg supplementation. The results obtained regarding the concentration of SCFAs are specific to each of the dietary groups tested which made it difficult to draw a robust conclusion, especially in the absence of any effect on the fecal microbiota from which they are derived. However, it is possible to observe that all three groups supplemented with Arg were characterized by a lower concentration of acetate, suggesting an involvement of Arg in the microbial production of these SCFAs, or in their absorption and utilization by the sows.

Finally, the present study showed that season could strongly influence both the productive performance of sows, and their physiological status and fecal microbiota.

In the cold season, the average weight of all the experimental groups was higher than in the warm season. It should be emphasized that season refers to the period during which the sows were gestating, so that piglets born from sows which gestated in the warm season grew in cold season and vice versa. This explains why piglets have a better growth performance in the cold season. During the warm season, the total number of piglets born decreased as compared with the cold season. As widely reported in the literature, this depends on the exposure of sows to suboptimal environmental conditions, such as high temperatures, which leads to heat stress, decreased productive performance and physiological alterations [74, 75]. Even if these aspects were not the main topic of the present study, the results herein obtained could be fundamental for the development of appropriate nutritional strategies in different environmental situations, leading to a nutritional approach with precision.

## Conclusion

In conclusion, the present study highlighted the effects of Arg administration during different periods of gestation. Specifically, the use of 38.3 g/d of Arg in the first third of gestation reduced the percentage of stillborn and LBW pigs, while its administration in the last third of pregnancy favored the percentage of pigs born with HBW and reduced the percentage of stillbirths. These differences could be attributed to the different metabolic fates of Arg during gestation and its involvement in placental efficiency. The present study emphasized the significant role of Arg in the physiology of pregnant sows and demonstrated the potential of metabolomics to elucidate physiological mechanisms. The complexity of Arg involvement in metabolic pathways was evident, so that partially contradicting outcomes hinder a straightforward explanation. Additional investigation is needed to fully understand Arg involvement during the gestation of sows.

## Supplementary Information

**Additional file 1: Table S1** Effect of Arg supplementation and season on the concentration of the identified metabolites in sow’s colostrum. **Table S2** Effect of Arg supplementation on the concentration of the identified metabolites of sows’ urine at d 35 of gestation. **Table S3** Effect of Arg supplementation on the identified metabolites of sow’ feces at d 35 of gestation. **Table S4** Effect of Arg supplementation and season on the identified metabolites concentrations in sows’ feces at d 106 of gestation. **Table S5** Microbial identifying differentially abundant taxa at d 35 of gestation in the two seasons. **Table S6** Microbial identifying differentially abundant taxa at d 106 of gestation in the two seasons. **Fig. S1** Effect of Arg supplementation on the metabolome characterization on the total colostrum spectra according to the seasons. **Fig. S2** Effect of Arg supplementation on the metabolites’ identification in sows’ feces at d 106 of gestation. **Fig. S3** Effect of the season on the metabolites’ identification in sows’ feces at d 106 of gestation. **Fig. S4** Beta diversity based on the season at d 35 (**A**) and d 106 (**B**) of gestation.

## Data Availability

The datasets used and/or analyzed during the current study are available from the corresponding author on reasonable request.

## Abbreviations

AAs: Amino acids
ADG: Average daily gain
Arg: Arginine
BW: Body weight
CV: Coefficient of variation
FI: Feed intake
HBW: High birthweight
HPLC: High-performance liquid chromatography
HS: Heat stress
Igs: Immunoglobulins
LBW: Low birthweight
NBW: Normal birthweight
NO: Nitric oxide
NRC: National research council
PC: Principal component
PLS-DA: Partial least squares-discriminant analysis
SCFAs: Short-chain fatty acids
VIP: Variable importance projection
