# Supplementary material for: Productive and metabolomic consequences of arginine supplementation in sows during different gestation periods in two different seasons
Source: J Anim Sci Biotechnol. 2024 Sep 19;15:121. doi: 10.1186/s40104-024-01079-4 (PMC11411819; doi:10.1186/s40104-024-01079-4)
Supplement: Supplementary file 1 — Additional file 1: Table S1 Effect of Arg supplementation and season on the concentration of the identified metabolites in sow’s colostrum. Table S2 Effect of Arg supplementation on the concentration of the identified metabolites of sows’ urine at d 35 of gestation. Table S3 Effect of Arg supplementation on the identified metabolites of sow’ feces at d 35 of gestation. Table S4 Effect of Arg supplementation and season on the identified metabolites concentrations in sows’ feces at d 106 of gestation. Table S5 Microbial identifying differentially abundant taxa at d 35 of gestation in the two seasons. Table S6 Microbial identifying differentially abundant taxa at d 106 of gestation in the two seasons. Fig. S1 Effect of Arg supplementation on the metabolome characterization on the total colostrum spectra according to the seasons. Fig. S2 Effect of Arg supplementation on the metabolites’ identification in sows’ feces at d 106 of gestation. Fig. S3 Effect of the season on the metabolites’ identification in sows’ feces at d 106 of gestation. Fig. S4 Beta diversity based on the season at d 35 (A) and d 106 (B) of gestation. [file 40104_2024_1079_MOESM1_ESM.docx]

**Additional file 1**

**Table S1** Effect of Arg supplementation and season on the concentration of the identified metabolites in sow’s colostrum

| **Metabolite, µmol/L** | **Diet^1^** | | | | | **Season^2^** | | | ***P*-value** | |
| --- | --- | --- | --- | --- | --- | --- | --- | --- | --- | --- |
|  | **CO** | **Early35** | **Late45** | **COM** | **SEM** | **Warm** | **Cold** | **SEM** | **Diet** | **Season** |
| Formate | 1.42^C^ | 1.44^C^ | 11.79^A^ | 3.80^B^ | 0.58 | 3.36 | 2.85 | 0.37 | < 0.001 | 0.225 |
| UMP | 720 | 677 | 623 | 706 | 60 | 719 | 643 | 44.0 | 0.659 | 0.221 |
| Uridine | 87.2^B^ | 65.0^C^ | 114.1^A^ | 83.8^B^ | 2.80 | 112.4^Y^ | 65.5^Z^ | 2.0 | < 0.001 | < 0.001 |
| cis Aconitate | 43.0 | 47.5 | 46.4 | 39.3 | 3.10 | 46.2 | 41.8 | 2.27 | 0.216 | 0.170 |
| UDP galactose | 1,846^B^ | 2,199^A^ | 1,756^C^ | 1,407^D^ | 12.5 | 1,562^Z^ | 2,027^Y^ | 9.0 | < 0.001 | < 0.001 |
| UDP glucuronate | 363^C^ | 257^D^ | 390^B^ | 416^A^ | 5.68 | 408^Y^ | 301^Z^ | 4.0 | < 0.001 | < 0.001 |
| UDP glucose | 1,019^ab^ | 1,297^a^ | 1,091^ab^ | 939^b^ | 99.0 | 965^Z^ | 1209^Y^ | 72.5 | 0.059 | 0.018 |
| UDP n-Acetylglucosamine | 428^A^ | 313^D^ | 363^C^ | 382^B^ | 5.7 | 365 | 373 | 4.1 | < 0.001 | 0.148 |
| Ribose | 62.8 | 62.2 | 63.3 | 59.9 | 2.30 | 64.2^y^ | 60.0^z^ | 1.75 | 0.716 | 0.078 |
| myo Inositol | 7,326 | 7,380 | 7,600 | 7,330 | 273 | 7,096^Z^ | 7,722^Y^ | 200 | 0.874 | 0.027 |
| Betaine | 470 | 496 | 369 | 449 | 44.7 | 476 | 416 | 32.8 | 0.199 | 0.200 |
| Taurine | 791 | 758 | 724 | 712 | 82.3 | 780 | 713 | 60.2 | 0.901 | 0.433 |
| Lactose | 72,232 | 69,152 | 70,111 | 73,610 | 2,592 | 69,674 | 72,879 | 1,900 | 0.596 | 0.233 |
| TMAO | 388 | 322 | 278 | 283 | 40.3 | 317 | 319 | 29.6 | 0.178 | 0.952 |
| sn Glycero-3-P-Choline | 2,368^C^ | 3,115^A^ | 2,902^B^ | 2,220^D^ | 15.0 | 2,756^Y^ | 2,501^Z^ | 11.1 | < 0.001 | < 0.001 |
| O Phosphocholine | 815 | 873 | 736 | 756 | 94.9 | 885^y^ | 705^z^ | 69.4 | 0.723 | 0.067 |
| Choline | 151^AB^ | 142^B^ | 159^A^ | 148^AB^ | 3.62 | 155^Y^ | 145^Z^ | 2.65 | 0.010 | 0.007 |
| Creatine phosphate | 410 | 347 | 351 | 300 | 30.0 | 354 | 350 | 23.5 | 0.105 | 0.909 |
| Creatine | 1,363 | 1,270 | 1,306 | 1,225 | 67.5 | 1,313 | 1,269 | 49.5 | 0.508 | 0.522 |
| Dimethylamine | 69.7 | 63.3 | 62.6 | 61.1 | 4.84 | 65.9 | 62.4 | 3.54 | 0.585 | 0.481 |
| Citrate | 675^B^ | 389^D^ | 937^A^ | 618^C^ | 7.61 | 742^Y^ | 525^Z^ | 5.44 | < 0.001 | < 0.001 |
| Carnitine | 194 | 211 | 177 | 175 | 23.8 | 170 | 209 | 17.9 | 0.669 | 0.108 |
| Succinate | 468^C^ | 545^B^ | 397^D^ | 574^A^ | 6.32 | 424^Z^ | 569^Y^ | 4.77 | < 0.001 | < 0.001 |
| Pyruvate | 224^B^ | 296^A^ | 147^C^ | 222^B^ | 4.24 | 206^Z^ | 225^Y^ | 3.15 | < 0.001 | < 0.001 |
| O Acetylcarnitine | 317^C^ | 362^B^ | 388^A^ | 277^D^ | 5.23 | 259^Z^ | 428^Y^ | 3.93 | < 0.001 | < 0.001 |
| Acetate | 82.3^C^ | 102.8^AB^ | 110.4^A^ | 98.5^B^ | 3.01 | 90.5^Z^ | 106.0^Y^ | 2.12 | < 0.001 | < 0.001 |
| Alanine | 19.7^B^ | 20.0^B^ | 19.5^B^ | 26.3^A^ | 1.41 | 20.8 | 21.6 | 0.99 | 0.001 | 0.528 |
| Lactate | 48.0^B^ | 45.3^B^ | 52.1^B^ | 109.3^A^ | 2.54 | 61.9^Y^ | 56.9^Z^ | 1.44 | < 0.001 | 0.025 |
| Valine | 8.06^B^ | 8.40^B^ | 13.33^A^ | 15.70^A^ | 1.00 | 11.9^y^ | 10.0^z^ | 0.71 | < 0.001 | 0.051 |

^1^ A total of 320 sows were divided into 4 experimental groups: CO, fed with a basal diet; Early35, fed with the CO diet with 21.8 g/d on-top Arg during the first 35 d of gestation; Late45, fed with the CO diet with 21.8 g/d on-top Arg during the last 45 d of gestation; COM, fed with the CO diet with 21.8 g/d on-top Arg during all gestation

^2^ The trial was performed during two seasons: warm, sows performed gestation between July and September; cold, sows performed gestation between November and January

Means within a row with different superscripts differ for diets’ contrasts ^ABCD^ *P* < 0.05 and ^abcd^ *P* < 0.01; for seasons’ contrast ^YZ^ *P* < 0.05 and ^yz^ *P* < 0.10

**Table S2** Effect of Arg supplementation on the concentration of the identified metabolites of sows’ urine at d 35 of gestation

| **Metabolite, µmol/L** | **Diet^1^** | | | ***P*-value** |
| --- | --- | --- | --- | --- |
|  | **CO** | **ARG** | **SEM** | **Diet** |
| 1-Methylnicotinamide | 16.9 | 16.0 | 4.10 | 0.880 |
| Formate | 315^B^ | 526^A^ | 57.2 | 0.010 |
| Hippurate | 5550^B^ | 6681^A^ | 31.7 | < 0.001 |
| Tyrosine | 320^B^ | 360^A^ | 7.54 | < 0.001 |
| 4-Hydroxyphenylacetate | 40.6^A^ | 30.0^B^ | 2.50 | 0.002 |
| trans-Aconitate | 71.6 | 82.0 | 15.5 | 0.647 |
| Cytosine | 8.85^B^ | 15.36^A^ | 1.43 | < 0.001 |
| Xanthosine | 61.8 | 46.5 | 10.2 | 0.300 |
| cis-Aconitate | 79.5^A^ | 51.5^B^ | 3.6 | < 0.001 |
| Allantoin | 641^A^ | 466^B^ | 10.10 | < 0.001 |
| Glucuronate | 311^A^ | 156^B^ | 6.55 | < 0.001 |
| Glucose | 63.2^B^ | 203.6^A^ | 4.59 | < 0.001 |
| Xylose | 14.6^B^ | 32.4^A^ | 2.05 | < 0.001 |
| Arabinose | 334^A^ | 312^B^ | 7.61 | 0.031 |
| Ascorbate | 119.4^A^ | 92.1^B^ | 4.27 | < 0.001 |
| Trigonelline | 36.8 | 43.2 | 6.74 | 0.507 |
| Tartrate | 135 | 125 | 4.7 | 0.161 |
| Gluconate | 85.5^A^ | 51.7^B^ | 3.5 | < 0.001 |
| Betaine | 116^B^ | 532^A^ | 6.83 | < 0.001 |
| Xylitol | 311 | 245 | 41.95 | 0.268 |
| Mannitol | 83.3^B^ | 98.0^A^ | 3.82 | 0.009 |
| N-Phenylacetylglycine | 1,548 | 1,241 | 148 | 0.149 |
| Glycine | 254^A^ | 158^B^ | 5.91 | < 0.001 |
| myo-Inositol | 257 | 244 | 43.3 | 0.844 |
| Methanol | 89.5 | 88.0 | 4.67 | 0.825 |
| Taurine | 971 | 691 | 147 | 0.185 |
| TMAO | 229^B^ | 416^A^ | 7.21 | < 0.001 |
| sn-Glycero-3-phosphocholine | 7.69 | 8.18 | 1.27 | 0.790 |
| Dimethyl sulfone | 54.4 | 43.7 | 8.29 | 0.370 |
| Creatinine | 8997 | 8158 | 1192 | 0.625 |
| Creatine | 226.1^A^ | 61.8^B^ | 5.1 | < 0.001 |
| N,N-Dimethylglycine | 37.6^B^ | 65.7^A^ | 5.34 | < 0.001 |
| Dimethylamine | 141 | 108 | 14.95 | 0.127 |
| Citrate | 46.9 | 51.3 | 10.12 | 0.762 |
| beta-Alanine | 106.3 | 91.5 | 12.45 | 0.410 |
| Glutamine | 38.6 | 43.0 | 2.63 | 0.249 |
| Succinate | 6.85 | 6.88 | 1.08 | 0.989 |
| Pyruvate | 4.06 | 3.85 | 0.298 | 0.623 |
| 5-Hydroxymethyl-4-methyluracil | 823^A^ | 573^B^ | 85.9 | 0.043 |
| 5-Aminopentanoate | 15.4^B^ | 24.1^A^ | 1.80 | 0.001 |
| Biotin | 89.6 | 89.1 | 3.87 | 0.927 |
| Sebacate | 50.2 | 49.7 | 7.86 | 0.924 |
| O-Acetylcarnitine | 3.77 | 5.72 | 1.4 | 0.333 |
| N-Acetylglucosamine | 33.7^A^ | 27.2^B^ | 2.29 | 0.040 |
| N-Acetylglutamate | 43.3^A^ | 34.4^B^ | 2.60 | 0.017 |
| N6-Acetyllysine | 19.0 | 15.9 | 1.73 | 0.215 |
| Acetate | 33.3^B^ | 46.8^A^ | 2.41 | < 0.001 |
| Tiglyglycine | 8.3 | 11.4 | 2.0 | 0.275 |
| Lysine | 396^A^ | 270^B^ | 7.63 | < 0.001 |
| Alanine | 26.3 | 26.8 | 4.81 | 0.944 |
| 2-Hydroxyisobutyrate | 35.9 | 34.8 | 3.84 | 0.844 |
| Lactate | 23.7^A^ | 12.3^B^ | 1.74 | < 0.001 |
| 3-Hydroxyisovalerate | 8.93 | 13.94 | 3.12 | 0.265 |
| Fucose | 41.1 | 32.2 | 5.66 | 0.275 |
| 3-Aminoisobutyrate | 4.38 | 5.51 | 1.92 | 0.684 |
| 2,3-Butanediol | 8.34^A^ | 5.44^B^ | 1.10 | 0.048 |
| Methylsuccinate | 11.15 | 8.83 | 1.31 | 0.202 |
| 3-Hydroxyisobutyrate | 7.59 | 9.30 | 2.05 | 0.564 |
| Leucine | 29.7^A^ | 17.5^B^ | 2.05 | < 0.001 |
| N-Isovaleroylglycine | 74.1^A^ | 53.4^B^ | 6.56 | 0.029 |
| Pantothenate | 24.5 | 20.1 | 1.96 | 0.107 |

^1^ A total of 320 sows were divided into 4 experimental groups: CO, fed with a basal diet; Early35, fed with the CO diet with 21.8 g/d on-top Arg during the first 35 d of gestation; Late45, fed with the CO diet with 21.8 g/d on-top Arg during the last 45 d of gestation; COM, fed with the CO diet with 21.8 g/d on-top Arg during all gestation. Urine samples collected were split into two different groups: CO (samples from sows belonging to groups CO and Late45) and ARG (samples from sows belonging to groups Early35 and COM)

Means within a row with different superscripts differ: ^AB^ *P* < 0.05; ^ab^ *P* < 0.10

**Table S3** Effect of Arg supplementation on the identified metabolites of sow’ feces at d 35 of gestation

| Metabolites, µmol/L | Diet ^1^ | | SEM | *P*-value |
| --- | --- | --- | --- | --- |
|  | **CO** | **ARG** |  | **Diet** |
| Formate | 1.08 | 1.06 | 0.47 | 0.974 |
| Hypoxanthine | 2.27 | 2.12 | 0.95 | 0.597 |
| Xanthine | 1.88 | 1.84 | 0.32 | 0.940 |
| Phenylalanine | 3.06 | 2.92 | 0.33 | 0.776 |
| Phenylacetate | 16.1 | 14.7 | 1.87 | 0.605 |
| 3-Phenylpropionate | 22.8 | 20.9 | 2.18 | 0.573 |
| Tyrosine | 2.49 | 2.32 | 0.24 | 0.630 |
| 3-Hydroxyphenylacetate | 6.53 | 5.80 | 0.99 | 0.641 |
| Fumarate | 0.518 | 0.733 | 0.39 | 0.704 |
| Uracil | 4.21^A^ | 2.24^B^ | 0.45 | 0.004 |
| Glucose | 12.3 | 12.3 | 1.68 | 0.991 |
| Galactose | 4.82^A^ | 3.81^B^ | 0.30 | 0.028 |
| Xylose | 9.87 | 7.43 | 1.85 | 0.392 |
| Arabinose | 5.95 | 4.62 | 0.90 | 0.338 |
| 1,3-Dihydroxyacetone | 0.432 | 0.382 | 0.273 | 0.902 |
| Lactate | 0.038 | 0.046 | 0.008 | 0.537 |
| Serine | 4.86 | 4.08 | 0.88 | 0.531 |
| Betaine | 0.669 | 0.936 | 0.15 | 0.249 |
| myo-Inositol | 3.54 | 3.97 | 0.24 | 0.242 |
| Threonine | 3.60 | 4.58 | 1.0 | 0.476 |
| Glycerol | 13.0 | 12.5 | 1.54 | 0.855 |
| Glycine | 2.90 | 3.55 | 0.48 | 0.377 |
| Methanol | 0.39^B^ | 0.53^A^ | 0.04 | 0.025 |
| Proline | 2.10 | 1.97 | 0.42 | 0.833 |
| Carnitine | 0.282 | 0.264 | 0.02 | 0.558 |
| Choline | 1.03 | 1.19 | 0.12 | 0.410 |
| Dimethyl sulfone | 0.303 | 0.384 | 0.06 | 0.403 |
| Malonate | 0.95 | 1.02 | 0.14 | 0.764 |
| Lysine | 5.86 | 5.82 | 0.75 | 0.968 |
| 2-Oxoglutarate | 1.17 | 1.47 | 0.56 | 0.729 |
| Aspartate | 7.60 | 8.33 | 0.79 | 0.552 |
| Methionine sulfoxide | 0.548 | 0.548 | 0.08 | 1.000 |
| Methionine | 2.42 | 2.16 | 0.69 | 0.796 |
| Succinate | 1.66 | 1.88 | 0.18 | 0.416 |
| Isobutyrate | 52.2 | 53.5 | 3.35 | 0.745 |
| Pyruvate | 0.005 | 0.013 | 0.008 | 0.484 |
| Glutamate | 11.0 | 13.0 | 1.52 | 0.404 |
| Acetoacetate | 0.791 | 0.840 | 0.14 | 0.823 |
| Acetoin | 2.26 | 2.20 | 0.31 | 0.907 |
| Levulinate | 3.03 | 3.01 | 0.34 | 0.962 |
| Acetate | 1059 | 1028 | 118 | 0.866 |
| Thymine | 1.18 | 1.19 | 0.21 | 0.977 |
| Alanine | 5.60 | 7.26 | 0.99 | 0.293 |
| Cadaverine | 7.23 | 5.61 | 1.00 | 0.313 |
| 2-Hydroxyisobutyrate | 0.579 | 0.579 | 0.35 | 0.999 |
| Valerate | 86.7 | 94.5 | 12.4 | 0.981 |
| Fucose | 0.668 | 0.987 | 0.29 | 0.475 |
| 3-Hydroxybutyrate | 3.54 | 2.79 | 0.37 | 0.187 |
| Isopropanol | 0.345 | 0.142 | 0.11 | 0.235 |
| 3-Methyl-2-oxovalerate | 0.479 | 0.430 | 0.11 | 0.771 |
| 2-Methyl-3-ketovalerate | 0.258 | 0.239 | 0.08 | 0.870 |
| Methylsuccinate | 1.42 | 1.33 | 0.08 | 0.440 |
| Propionate | 517 | 532 | 62.5 | 0.877 |
| Isoleucine | 2.25 | 2.42 | 0.33 | 0.728 |
| Valine | 2.56 | 3.18 | 0.46 | 0.391 |
| Leucine | 3.66 | 3.69 | 0.41 | 0.960 |
| 2-Oxoisocaproate | 0.87^b^ | 1.12^a^ | 0.09 | 0.071 |
| Isovalerate | 34.2 | 33.0 | 4.0 | 0.847 |
| Butyrate | 181 | 180 | 24.0 | 0.965 |

^1^ A total of 320 sows were divided into 4 experimental groups: CO, fed with a basal diet; Early35, fed with the CO diet with 21.8 g/d on-top Arg during the first 35 d of gestation; Late45, fed with the CO diet with 21.8 g/d on-top Arg during the last 45 d of gestation; COM, fed with the CO diet with 21.8 g/d on-top Arg during all gestation. Urine samples collected were split into two different groups: CO (samples from sows belonging to groups CO and Late45) and ARG (samples from sows belonging to groups Early35 and COM)

Means within a row with different superscripts differ: ^AB^ *P* < 0.05; ^ab^ *P* < 0.10

**Table S4** Effect of Arg supplementation and season on the identified metabolites concentrations in sows’ feces at d 106 of gestation

| **Metabolite, µmol/L** | **Diet^1^** | | | | | **Season^2^** | | | ***P*-value** | |
| --- | --- | --- | --- | --- | --- | --- | --- | --- | --- | --- |
|  | **CO** | **Early35** | **Late45** | **COM** | **SEM** | **warm** | **cold** | **SEM** | **Diet** | **Season** |
| Formate | 0.618 | 0.517 | 0.536 | 0.479 | 0.07 | 0.509 | 0.566 | 0.05 | 0.597 | 0.448 |
| Hypoxanthine | 2.20 | 2.64 | 2.59 | 2.66 | 0.27 | 2.15^Z^ | 2.90^Y^ | 0.19 | 0.578 | 0.007 |
| Xanthine | 2.09 | 1.96 | 2.18 | 2.15 | 0.44 | 1.84 | 2.38 | 0.32 | 0.983 | 0.241 |
| Phenylalanine | 4.01 | 3.69 | 3.76 | 3.64 | 0.57 | 3.94 | 3.61 | 0.42 | 0.972 | 0.580 |
| Phenylacetate | 11.0 | 13.3 | 11.0 | 11.1 | 1.0 | 13.1^Y^ | 10.2^Z^ | 0.74 | 0.310 | 0.005 |
| 3-Phenylpropionate | 11.4 | 13.6 | 12.2 | 14.5 | 1.07 | 12.9 | 12.9 | 0.76 | 0.183 | 0.991 |
| Tyrosine | 2.61 | 2.24 | 2.24 | 2.29 | 0.46 | 2.49 | 2.20 | 0.35 | 0.924 | 0.538 |
| 3-Hydroxyphenylacetate | 3.43 | 3.45 | 3.29 | 4.30 | 0.58 | 4.16^y^ | 3.11^z^ | 0.39 | 0.598 | 0.071 |
| Fumarate | 0.333 | 0.293 | 0.356 | 0.272 | 0.03 | 0.255^Z^ | 0.372^Y^ | 0.02 | 0.198 | < 0.001 |
| Uracil | 4.28 | 4.53 | 4.61 | 4.44 | 0.44 | 3.91^Z^ | 5.02^Y^ | 0.32 | 0.953 | 0.014 |
| Glucose | 8.61 | 9.66 | 10.00 | 10.27 | 0.90 | 9.43 | 9.80 | 0.67 | 0.568 | 0.701 |
| Galactose | 2.79 | 2.70 | 2.24 | 2.65 | 0.24 | 2.48 | 2.71 | 0.18 | 0.384 | 0.365 |
| Xylose | 5.69 | 5.57 | 5.45 | 4.83 | 0.48 | 5.50 | 5.26 | 0.36 | 0.610 | 0.630 |
| Arabinose | 3.19 | 3.58 | 2.72 | 3.11 | 0.53 | 3.30 | 2.98 | 0.38 | 0.718 | 0.558 |
| 1,3-Dihydroxyacetone | 0.255 | 0.287 | 0.260 | 0.266 | 0.03 | 0.268 | 0.266 | 0.02 | 0.847 | 0.926 |
| Lactate | 1.64 | 3.13 | 3.02 | 2.17 | 0.85 | 1.59^Z^ | 3.39^Y^ | 0.61 | 0.551 | 0.040 |
| Serine | 5.54 | 5.06 | 5.14 | 4.97 | 0.38 | 5.18 | 5.18 | 0.28 | 0.706 | 0.995 |
| Betaine | 0.853 | 0.864 | 0.894 | 0.829 | 0.08 | 0.732^Z^ | 0.988^Y^ | 0.06 | 0.956 | 0.003 |
| myo-Inositol | 2.95 | 2.44 | 2.97 | 2.48 | 0.25 | 2.47^z^ | 2.95^y^ | 0.19 | 0.260 | 0.071 |
| Threonine | 5.95 | 5.48 | 5.74 | 6.15 | 0.26 | 6.12^Y^ | 5.54^Z^ | 0.19 | 0.267 | 0.029 |
| Glycerol | 6.82 | 8.67 | 7.69 | 8.55 | 0.76 | 8.10 | 7.77 | 0.56 | 0.274 | 0.674 |
| Glycine | 7.53 | 6.43 | 7.11 | 7.21 | 0.78 | 7.07 | 7.04 | 0.58 | 0.769 | 0.969 |
| Methanol | 0.787 | 0.876 | 0.926 | 0.872 | 0.29 | 0.890 | 0.838 | 0.21 | 0.990 | 0.858 |
| Proline | 4.33 | 3.65 | 4.01 | 3.95 | 0.45 | 4.13 | 3.84 | 0.32 | 0.726 | 0.502 |
| Carnitine | 0.149 | 0.161 | 0.188 | 0.145 | 0.02 | 0.163 | 0.158 | 0.01 | 0.176 | 0.762 |
| Choline | 0.629 | 0.649 | 0.613 | 0.580 | 0.04 | 0.589 | 0.646 | 0.03 | 0.590 | 0.140 |
| Dimethyl sulfone | 0.520 | 0.486 | 0.412 | 0.491 | 0.21 | 0.522 | 0.433 | 0.16 | 0.986 | 0.683 |
| Malonate | 1.04 | 1.58 | 1.27 | 1.48 | 0.34 | 1.53 | 1.15 | 0.25 | 0.681 | 0.274 |
| Lysine | 6.74 | 7.63 | 7.37 | 7.47 | 0.28 | 7.16 | 7.45 | 0.20 | 0.121 | 0.317 |
| 2-Oxoglutarate | 2.07 | 1.93 | 1.82 | 2.22 | 0.18 | 2.05 | 1.97 | 0.14 | 0.395 | 0.662 |
| Aspartate | 6.56 | 5.75 | 6.54 | 5.94 | 0.51 | 6.04 | 6.36 | 0.38 | 0.563 | 0.548 |
| Methionine sulfoxide | 0.640 | 0.534 | 0.604 | 0.625 | 0.05 | 0.601 | 0.601 | 0.03 | 0.434 | 0.999 |
| Methionine | 2.39 | 2.34 | 2.28 | 2.18 | 0.44 | 2.31 | 2.28 | 0.33 | 0.989 | 0.938 |
| Succinate | 3.13 | 2.24 | 2.85 | 3.17 | 0.50 | 3.16 | 2.53 | 0.38 | 0.507 | 0.230 |
| Isobutyrate | 36.0 | 41.8 | 40.5 | 44.4 | 4.20 | 47.3^Y^ | 34.1^Z^ | 3.10 | 0.545 | 0.002 |
| Pyruvate | 0.575 | 0.383 | 0.640 | 0.433 | 0.22 | 0.429 | 0.576 | 0.16 | 0.795 | 0.491 |
| Glutamate | 16.2 | 15.7 | 17.1 | 15.6 | 1.17 | 15.1^z^ | 17.2^y^ | 0.85 | 0.780 | 0.078 |
| Acetoacetate | 0.762 | 0.879 | 0.915 | 0.886 | 0.08 | 0.954^Y^ | 0.767^Z^ | 0.06 | 0.485 | 0.017 |
| Acetoin | 2.30 | 2.25 | 2.11 | 1.88 | 0.44 | 2.85^Y^ | 1.59^Z^ | 0.30 | 0.907 | 0.007 |
| Levulinate | 2.42 | 2.13 | 2.43 | 2.06 | 0.46 | 2.55 | 1.99 | 0.34 | 0.917 | 0.246 |
| Acetate | 547 | 746 | 701 | 668 | 81.5 | 720 | 611 | 59.7 | 0.337 | 0.198 |
| Thymine | 1.86 | 1.95 | 1.81 | 1.87 | 0.22 | 1.49^Y^ | 2.25^Z^ | 0.16 | 0.972 | < 0.001 |
| Alanine | 17.0 | 16. | 17.6 | 16.1 | 1.32 | 16.0 | 17.7 | 1.0 | 0.896 | 0.225 |
| Cadaverine | 5.33 | 6.56 | 5.41 | 6.36 | 0.77 | 6.83^Y^ | 5.00^Z^ | 0.56 | 0.552 | 0.022 |
| 2-Hydroxyisobutyrate | 0.360 | 0.404 | 0.403 | 0.383 | 0.05 | 0.403 | 0.372 | 0.04 | 0.911 | 0.544 |
| Valerate | 62.6 | 73.6 | 62.1 | 68.7 | 6.66 | 72.8^y^ | 60.7^z^ | 4.90 | 0.557 | 0.078 |
| Fucose | 2.23 | 2.02 | 1.47 | 2.12 | 0.29 | 2.20^y^ | 1.72^z^ | 0.20 | 0.238 | 0.099 |
| 3-Hydroxybutyrate | 1.92 | 2.34 | 2.77 | 1.71 | 0.46 | 2.62^y^ | 1.76^z^ | 0.34 | 0.348 | 0.057 |
| Ethanol | 0.849 | 1.081 | 0.759 | 1.125 | 0.39 | 0.982 | 0.901 | 0.21 | 0.770 | 0.782 |
| Isopropanol | 0.602 | 0.956 | 1.086 | 1.022 | 0.27 | 0.736 | 1.085 | 0.21 | 0.534 | 0.227 |
| 3-Methyl-2-oxovalerate | 1.39 | 1.17 | 1.20 | 1.56 | 0.20 | 1.38 | 1.28 | 0.15 | 0.448 | 0.610 |
| 2-Methyl-3-ketovalerate | 0.830 | 0.809 | 0.848 | 0.912 | 0.28 | 0.837 | 0.861 | 0.21 | 0.994 | 0.936 |
| Methylsuccinate | 0.840 | 1.024 | 1.060 | 0.914 | 0.30 | 1.107 | 0.825 | 0.22 | 0.950 | 0.348 |
| Propionate | 268 | 372 | 372 | 353 | 41.0 | 342 | 341 | 29.0 | 0.179 | 0.642 |
| Isoleucine | 5.77 | 5.73 | 5.71 | 5.18 | 0.69 | 5.56 | 5.62 | 0.51 | 0.915 | 0.928 |
| Valine | 7.91 | 7.90 | 8.17 | 7.24 | 0.81 | 7.47 | 8.13 | 0.60 | 0.858 | 0.437 |
| 2-Aminobutyrate | 0.624 | 0.523 | 0.438 | 0.451 | 0.21 | 0.482 | 0.527 | 0.15 | 0.917 | 0.837 |
| Leucine | 6.67 | 6.76 | 6.65 | 6.05 | 0.74 | 6.43 | 6.61 | 0.55 | 0.896 | 0.817 |
| 2-Oxoisocaproate | 1.75 | 1.32 | 1.77 | 1.74 | 0.36 | 1.46 | 1.83 | 0.28 | 0.770 | 0.341 |
| Isovalerate | 23.8 | 28.4 | 25.0 | 29.0 | 3.35 | 30.9^Y^ | 22.3^Z^ | 2.45 | 0.615 | 0.013 |
| Butyrate | 98.1 | 157.7 | 121.8 | 151.1 | 21.1 | 140 | 124 | 15.3 | 0.153 | 0.459 |

^1^ A total of 320 sows were divided into 4 experimental groups: CO, fed with a basal diet; Early35, fed with the CO diet with 21.8 g/d on-top Arg during the first 35 d of gestation; Late45, fed with the CO diet with 21.8 g/d on-top Arg during the last 45 d of gestation; COM, fed with the CO diet with 21.8 g/d on-top Arg during all gestation

^2^ The trial was performed during two seasons: warm, sows performed gestation between July and September; cold, sows performed gestation between November and January

Means within a row with different superscripts differ for diets’ contrasts ^ABCD^ *P* < 0.05 and ^abcd^ *P* < 0.01; for seasons’ contrast ^YZ^ *P* < 0.05 and ^yz^ *P* < 0.10

**Table S5** Microbial identifying differentially abundant taxa at d 35 of gestation in the two seasons

| **Marker** | **Species** | **Season** | **ef_lda** | ***P*-value** | ***P*_adj_** |
| --- | --- | --- | --- | --- | --- |
| marker1 | Clostridium sensu< stricto 1_s__ | W | 4.60812 | 1.80E-02 | 1.80E-02 |
| marker2 | Christensenellaceae R-7 group_s__ | W | 3.78504 | 3.54E-02 | 3.54E-02 |
| marker3 | Romboutsia_s__ | W | 3.77638 | 2.78E-02 | 2.78E-02 |
| marker4 | butyricum | W | 3.65512 | 1.80E-02 | 1.80E-02 |
| marker5 | Rikenellaceae RC9 gut group_s__ | W | 3.53252 | 1.55E-02 | 1.55E-02 |
| marker6 | Ruminococcus_s__ | W | 3.48232 | 4.93E-02 | 4.93E-02 |
| marker7 | Lachnospiraceae_g___s__ | W | 3.48116 | 1.94E-02 | 1.94E-02 |
| marker8 | Lachnospiraceae NK4A136 group_s__ | W | 3.19379 | 2.38E-03 | 2.38E-03 |
| marker9 | disporicum | W | 3.18565 | 4.33E-02 | 4.33E-02 |
| marker10 | Phascolarctobacterium_s__ | W | 3.00511 | 3.56E-03 | 3.56E-03 |
| marker11 | Lachnospiraceae AC2044 group_s__ | W | 2.90758 | 2.33E-02 | 2.33E-02 |
| marker12 | Paludibacteraceae_g___s__ | W | 2.83871 | 1.12E-03 | 1.12E-03 |
| marker13 | dgA-11 gut group_s__ | W | 2.58633 | 2.73E-02 | 2.73E-02 |
| marker14 | Rhodospirillales_f___g___s__ | W | 2.23604 | 3.08E-03 | 3.08E-03 |
| marker15 | Ligilactobacillus_s__ | W | 2.19777 | 2.37E-02 | 2.37E-02 |
| marker16 | p-1088-a5 gut group_s__ | W | 2.00954 | 3.73E-03 | 3.73E-03 |
| marker17 | Streptococcus_s__ | C | 5.05039 | 7.96E-05 | 7.96E-05 |
| marker18 | alactolyticus | C | 3.86835 | 1.63E-04 | 1.63E-04 |
| marker19 | pycnus | C | 3.0595 | 2.14E-03 | 2.14E-03 |
| marker20 | ventriculi | C | 2.69346 | 1.67E-02 | 1.67E-02 |
| marker21 | [Eubacterium] hallii group_s__ | C | 2.66465 | 3.20E-02 | 3.20E-02 |
| marker22 | Alloprevotella_s__ | C | 2.46134 | 1.15E-02 | 1.15E-02 |
| marker23 | Lactobacillales_f___g___s__ | C | 2.09128 | 4.18E-02 | 4.18E-02 |
| marker24 | Erysipelotrichaceae UCG-003_s__ | C | 2.03806 | 2.17E-02 | 2.17E-02 |

The seasons included W (warm) and C (cold)

**Table S6** Microbial identifying differentially abundant taxa at d 106 of gestation in the two seasons

| **Marker** | **Species** | **Season** | **ef_lda** | ***P*-value** | ***P*_adj_** |
| --- | --- | --- | --- | --- | --- |
| marker1 | Christensenellaceae R-7 group_s__ | W | 4.313293 | 8.83E-05 | 8.83E-05 |
| marker2 | UCG-002_s__ | W | 4.16999 | 5.95E-05 | 5.95E-05 |
| marker3 | Methanobrevibacter_s__ | W | 3.924161 | 4.35E-04 | 4.35E-04 |
| marker4 | Clostridia UCG-014_f___g___s__ | W | 3.862944 | 1.51E-02 | 1.51E-02 |
| marker5 | Escherichia-Shigella_s__ | W | 3.856009 | 8.81E-06 | 8.81E-06 |
| marker6 | [Eubacterium] coprostanoligenesgroup_g___s__ | W | 3.829545 | 4.73E-05 | 4.73E-05 |
| marker7 | tertium | W | 3.813002 | 4.60E-03 | 4.60E-03 |
| marker8 | Romboutsia_s__ | W | 3.781248 | 3.62E-02 | 3.62E-02 |
| marker9 | UCG-010_g___s__ | W | 3.484085 | 3.39E-02 | 3.39E-02 |
| marker10 | Christensenellaceae_g___s__ | W | 3.458016 | 1.52E-02 | 1.52E-02 |
| marker11 | Lachnospiraceae XPB1014 group_s__ | W | 3.318315 | 1.68E-02 | 1.68E-02 |
| marker12 | plebeius | W | 3.309414 | 2.01E-02 | 2.01E-02 |
| marker13 | RF39_f___g___s__ | W | 3.260888 | 8.35E-05 | 8.35E-05 |
| marker14 | Clostridia vadinBB60 group_f___g___s__ | W | 3.260777 | 1.03E-02 | 1.03E-02 |
| marker15 | togonis | W | 3.18529 | 7.29E-04 | 7.29E-04 |
| marker16 | cecorum | W | 3.142775 | 4.12E-02 | 4.12E-02 |
| marker17 | champanellensis | W | 3.129775 | 4.33E-03 | 4.33E-03 |
| marker18 | Oscillospirales_f___g___s__ | W | 3.119563 | 1.33E-03 | 1.33E-03 |
| marker19 | ventriculi | W | 3.110506 | 4.59E-02 | 4.59E-02 |
| marker20 | choerinum | W | 3.006581 | 1.36E-02 | 1.36E-02 |
| marker21 | Turicibacter_s__ | W | 2.985012 | 3.34E-06 | 3.34E-06 |
| marker22 | Candidatus Soleaferrea_s__ | W | 2.944798 | 1.75E-02 | 1.75E-02 |
| marker23 | Monoglobus_s__ | W | 2.92382 | 1.07E-02 | 1.07E-02 |
| marker24 | delbrueckii | W | 2.788345 | 2.04E-04 | 2.04E-04 |
| marker25 | Limosilactobacillus_s__ | W | 2.78147 | 4.15E-04 | 4.15E-04 |
| marker26 | Acetitomaculum_s__ | W | 2.763379 | 1.74E-03 | 1.74E-03 |
| marker27 | hyointestinalis | W | 2.558173 | 6.78E-05 | 6.78E-05 |
| marker28 | Clostridia_o___f___g___s__ | W | 2.545523 | 1.68E-04 | 1.68E-04 |
| marker29 | vulgatus | W | 2.528135 | 1.06E-03 | 1.06E-03 |
| marker30 | Papillibacter_s__ | W | 2.474783 | 1.18E-03 | 1.18E-03 |
| marker31 | Peptococcaceae_g___s__ | W | 2.439123 | 2.56E-03 | 2.56E-03 |
| marker32 | RumEn M2_s__ | W | 2.431272 | 1.48E-02 | 1.48E-02 |
| marker33 | [Eubacterium] saphenum group_s__ | W | 2.399823 | 3.40E-03 | 3.40E-03 |
| marker34 | Izemoplasmatales_f___g___s__ | W | 2.392974 | 5.80E-03 | 5.80E-03 |
| marker35 | Actinomyces_s__ | W | 2.339013 | 1.18E-03 | 1.18E-03 |
| marker36 | [Anaerorhabdus] furcosa group_s__ | W | 2.234251 | 3.56E-02 | 3.56E-02 |
| marker37 | Pyramidobacter_s__ | W | 2.180416 | 3.41E-02 | 3.41E-02 |
| marker38 | [Ruminococcus] torques group_s__ | W | 2.160879 | 4.67E-02 | 4.67E-02 |
| marker39 | variabile | W | 2.110314 | 1.38E-03 | 1.38E-03 |
| marker40 | Cerasicoccus_s__ | W | 2.098492 | 3.24E-02 | 3.24E-02 |
| marker41 | Candidatus Stoquefichus_s__ | W | 2.052589 | 1.21E-02 | 1.21E-02 |
| marker42 | Citrobacter_s__ | W | 2.046529 | 1.62E-02 | 1.62E-02 |
| marker43 | Butyricimonas_s__ | W | 2.020578 | 3.69E-03 | 3.69E-03 |
| marker44 | Lachnospiraceae UCG-010_s__ | W | 2.000044 | 1.19E-02 | 1.19E-02 |
| marker45 | Streptococcus_s__ | C | 5.090741 | 2.24E-08 | 2.24E-08 |
| marker46 | HT002_s__ | C | 4.384758 | 1.87E-06 | 1.87E-06 |
| marker47 | butyricum | C | 3.630122 | 3.06E-02 | 3.06E-02 |
| marker48 | alactolyticus | C | 3.609553 | 1.94E-08 | 1.94E-08 |
| marker49 | Prevotellaceae NK3B31 group_s__ | C | 3.544073 | 4.40E-02 | 4.40E-02 |
| marker50 | Prevotella_s__ | C | 3.537332 | 1.07E-02 | 1.07E-02 |
| marker51 | Methanosphaera_s__ | C | 3.533622 | 2.01E-03 | 2.01E-03 |
| marker52 | p-251-o5_g___s__ | C | 3.39225 | 2.58E-02 | 2.58E-02 |
| marker53 | Fibrobacter_s__ | C | 3.229276 | 1.03E-05 | 1.03E-05 |
| marker54 | Muribaculaceae_g___s__ | C | 3.22158 | 6.68E-03 | 6.68E-03 |
| marker55 | Selenomonadaceae_g___s__ | C | 3.124566 | 6.29E-03 | 6.29E-03 |
| marker56 | Prevotellaceae UCG-003_s__ | C | 3.101741 | 2.87E-03 | 2.87E-03 |
| marker57 | flavefaciens | C | 3.098443 | 1.02E-02 | 1.02E-02 |
| marker58 | disporicum | C | 3.082411 | 3.99E-02 | 3.99E-02 |
| marker59 | Corynebacterium_s__ | C | 3.079656 | 1.47E-02 | 1.47E-02 |
| marker60 | dgA-11 gut group_s__ | C | 2.930317 | 1.25E-02 | 1.25E-02 |
| marker61 | Roseburia_s__ | C | 2.911227 | 1.39E-02 | 1.39E-02 |
| marker62 | Lachnospiraceae UCG-007_s__ | C | 2.778974 | 3.92E-02 | 3.92E-02 |
| marker63 | catus | C | 2.675361 | 4.40E-03 | 4.40E-03 |
| marker64 | Bacteria_p___c___o___f___g___s__ | C | 2.662817 | 9.63E-03 | 9.63E-03 |
| marker65 | Streptococcaceae_g___s__ | C | 2.642712 | 2.78E-03 | 2.78E-03 |
| marker66 | Ligilactobacillus_s__ | C | 2.629705 | 7.85E-04 | 7.85E-04 |
| marker67 | prausnitzii | C | 2.598476 | 4.03E-03 | 4.03E-03 |
| marker68 | Butyricicoccus_s__ | C | 2.589847 | 5.03E-03 | 5.03E-03 |
| marker69 | Prevotella_7_s__ | C | 2.581019 | 4.10E-02 | 4.10E-02 |
| marker70 | Lachnoclostridium_s__ | C | 2.530305 | 9.62E-03 | 9.62E-03 |
| marker71 | Butyricicoccaceae_g___s__ | C | 2.49178 | 3.93E-02 | 3.93E-02 |
| marker72 | Rhodococcus_s__ | C | 2.414192 | 2.78E-03 | 2.78E-03 |
| marker73 | Lachnospiraceae NK4B4 group_s__ | C | 2.387078 | 3.54E-02 | 3.54E-02 |
| marker74 | Shuttleworthia_s__ | C | 2.296563 | 4.81E-02 | 4.81E-02 |
| marker75 | Succinivibrionaceae UCG-001_s__ | C | 2.257574 | 1.08E-02 | 1.08E-02 |
| marker76 | Akkermansia_s__ | C | 2.244781 | 3.05E-04 | 3.05E-04 |
| marker77 | T34_g___s__ | C | 2.230921 | 6.98E-04 | 6.98E-04 |
| marker78 | obeum | C | 2.167063 | 2.10E-02 | 2.10E-02 |
| marker79 | CAG-873_s__ | C | 2.117961 | 9.71E-03 | 9.71E-03 |
| marker80 | Dietzia_s__ | C | 2.100879 | 1.08E-02 | 1.08E-02 |
| marker81 | bornimense | C | 2.098241 | 4.01E-02 | 4.01E-02 |
| marker82 | Clostridiaceae_g___s__ | C | 2.090023 | 1.98E-02 | 1.98E-02 |
| marker83 | Schwartzia_s__ | C | 2.072611 | 5.08E-04 | 5.08E-04 |

The seasons included W (warm) and C (cold)


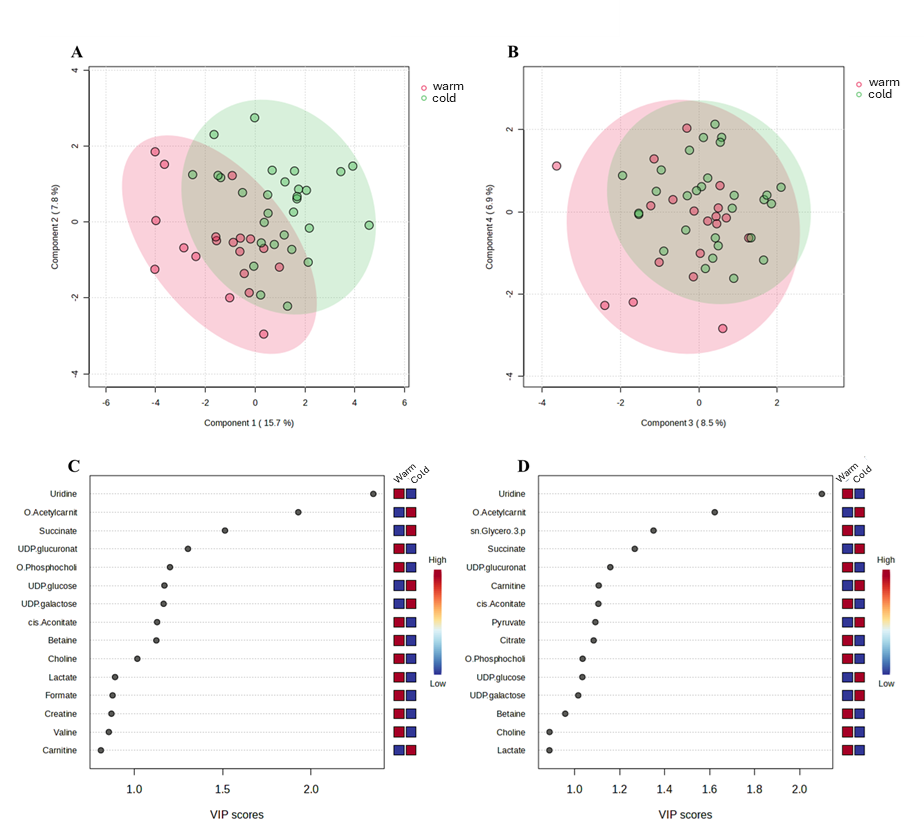


**Fig. S1** Effect of Arg supplementation on the metabolome characterization on the total colostrum spectra according to the seasons. A total of 320 sows were divided during 2 seasons (warm vs cold) into 4 experimental groups: CO, fed with a basal diet; Early35, fed with the CO diet with 21.8 g/d on-top Arg during the first 35 d of gestation; Late45, fed with the CO diet with 21.8 g/d on-top Arg during the last 45 d of gestation; COM, fed with the CO diet with 21.8 g/d on-top Arg during all gestation. PLS-DA was applied to investigate differences in the metabolome profile according to the season. Score plots of the PC were reported. Although the overlapping of the seasons, a separation between them under the PC2 can be observed (**A**)


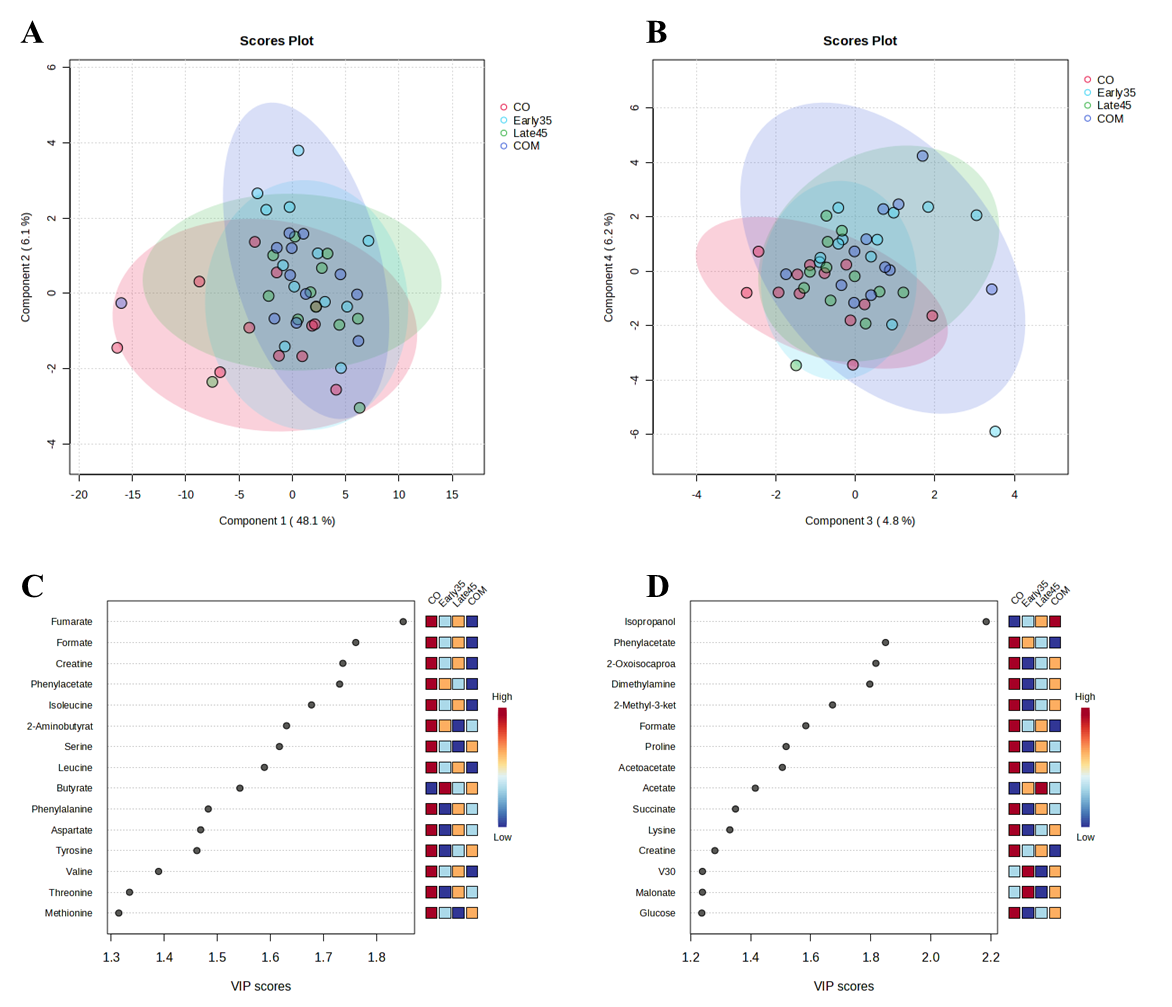


**Fig. S2** Effect of Arg supplementation on the metabolites’ identification in sows’ feces at d 106 of gestation. A total of 320 sows were divided into 4 experimental groups: CO, fed with a basal diet; Early35, fed with the CO diet with 21.8 g/d on-top Arg during the first 35 d of gestation; Late45, fed with the CO diet with 21.8 g/d on-top Arg during the last 45 d of gestation; COM, fed with the CO diet with 21.8 g/d on-top Arg during all gestation. PLS-DA was applied to investigate differences in the metabolome profile according to the experimental group. Score plots of the PC were reported. No differences were observed in any of the components (**A** and **B**)

**
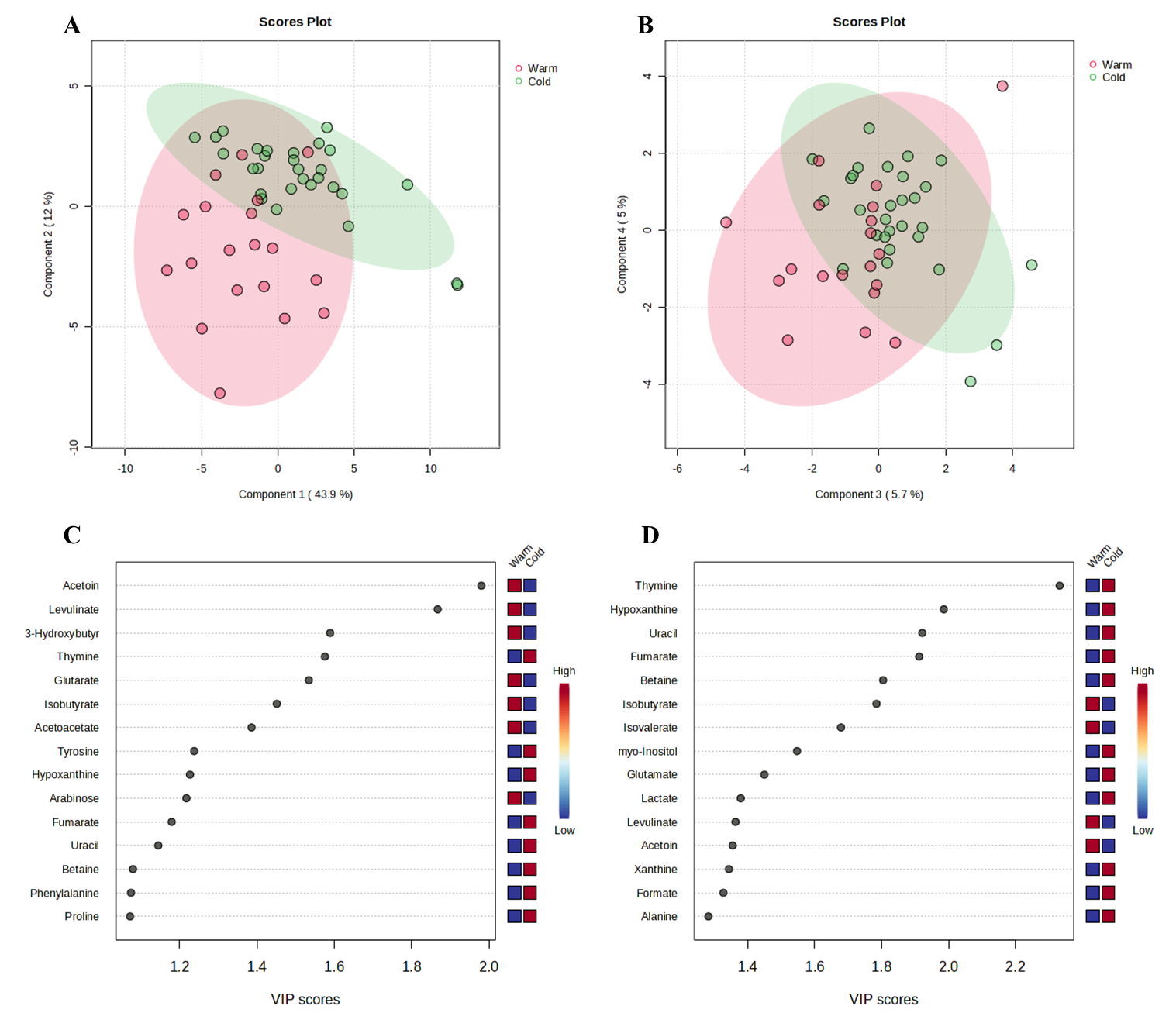
**

**Fig. S3** Effect of the season on the metabolites’ identification in sows’ feces at d 106 of gestation. A total of 320 sows were divided during 2 seasons (warm vs cold) into 4 experimental groups: CO, fed with a basal diet; Early35, fed with the CO diet with 21.8 g/d on-top Arg during the first 35 d of gestation; Late45, fed with the CO diet with 21.8 g/d on-top Arg during the last 45 d of gestation; COM, fed with the CO diet with 21.8 g/d on-top Arg during all gestation. PLS-DA was applied to investigate differences in the metabolome profile according to the season. Score plots of the PC1 were reported

**
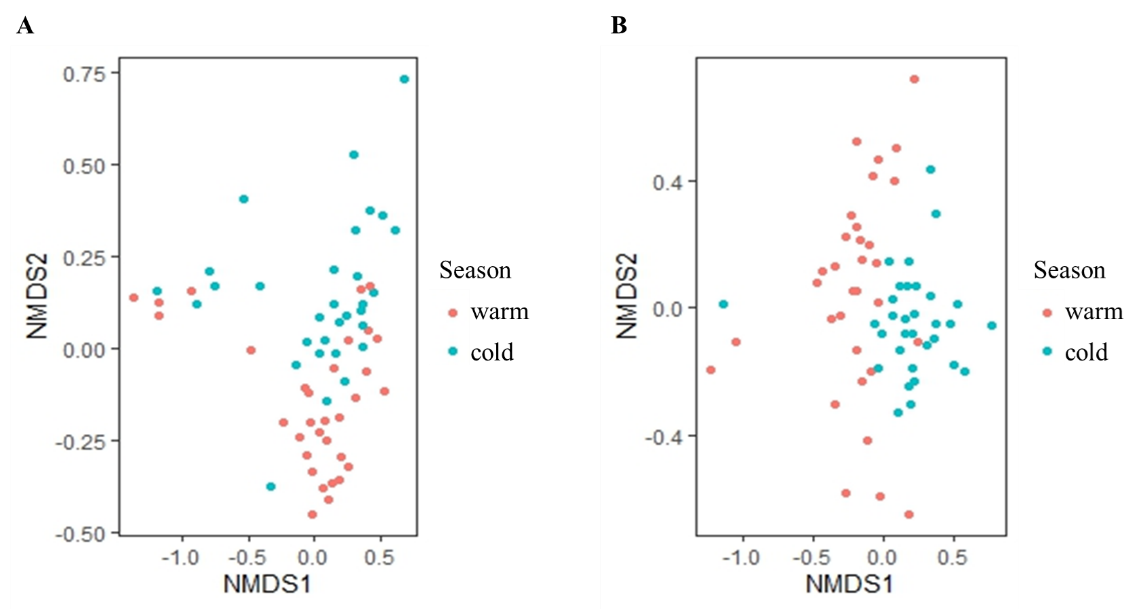
**

**Fig. S4** Beta diversity based on the season at d 35 (**A**) and d 106 (**B**) of gestation. A total of 320 sows were divided during 2 seasons (warm vs cold) into 4 experimental groups: CO, fed with a basal diet; Early35, fed with the CO diet with 21.8 g/d on-top Arg during the first 35 d of gestation; Late45, fed with the CO diet with 21.8 g/d on-top Arg during the last 45 d of gestation; COM, fed with the CO diet with 21.8 g/d on-top Arg during all gestation**.** Beta diversity was affected by the season at d 35 (**A**, R^2^ = 0.061; *P* = 0.002) and at d 106 (**B**, R^2^ = 0.104; *P* < 0.001)
